# Identification and Characterization of microRNAs from Peanut (*Arachis hypogaea* L.) by High-Throughput Sequencing

**DOI:** 10.1371/journal.pone.0027530

**Published:** 2011-11-16

**Authors:** Xiaoyuan Chi, Qingli Yang, Xiaoping Chen, Jinyan Wang, Lijuan Pan, Mingna Chen, Zhen Yang, Yanan He, Xuanqiang Liang, Shanlin Yu

**Affiliations:** 1 Shandong Peanut Research Institute, Qingdao, People's Republic of China; 2 Crops Research Institute, Guangdong Academy of Agricultural Sciences, People's Republic of China; 3 State Key Laboratory of Crop Genetics and Germplasm Enhancement, College of Horticulture, Nanjing Agricultural University, Nanjing, People's Republic of China; University of Pennsylvania School of Medicine, United States of America

## Abstract

**Background:**

MicroRNAs (miRNAs) are noncoding RNAs of approximately 21 nt that regulate gene expression in plants post-transcriptionally by endonucleolytic cleavage or translational inhibition. miRNAs play essential roles in numerous developmental and physiological processes and many of them are conserved across species. Extensive studies of miRNAs have been done in a few model plants; however, less is known about the diversity of these regulatory RNAs in peanut (Arachis hypogaea L.), one of the most important oilseed crops cultivated worldwide.

**Results:**

A library of small RNA from peanut was constructed for deep sequencing. In addition to 126 known miRNAs from 33 families, 25 novel peanut miRNAs were identified. The miRNA* sequences of four novel miRNAs were discovered, providing additional evidence for the existence of miRNAs. Twenty of the novel miRNAs were considered to be species-specific because no homolog has been found for other plant species. qRT-PCR was used to analyze the expression of seven miRNAs in different tissues and in seed at different developmental stages and some showed tissue- and/or growth stage-specific expression. Furthermore, potential targets of these putative miRNAs were predicted on the basis of the sequence homology search.

**Conclusions:**

We have identified large numbers of miRNAs and their related target genes through deep sequencing of a small RNA library. This study of the identification and characterization of miRNAs in peanut can initiate further study on peanut miRNA regulation mechanisms, and help toward a greater understanding of the important roles of miRNAs in peanut.

## Introduction

MicroRNAs (miRNA) are endogenous tiny RNAs (∼21 nt in length) that can play important regulatory roles in animals and plants by targeting mRNAs for cleavage or translational repression. Since the discovery of the first miRNA, lin 4 in Caenorhabditis elegans [1], thousands of miRNAs have been identified in various multi-cellular eukaryotes, including humans, flies, nematodes and plants, and are deposited in the miRBase database (http://www.mirbase.org/, Release 16.0, September 2010) [2], [3], [4]. There is increasing evidence that miRNAs play significant roles in various biological processes, including developmental transition and patterning, response to the environment and maintaining genome stability as well as defense against viruses and bacteria in eukaryotes. Although interest in miRNAs has attracted the attention of many scientists, and hundreds of plant miRNAs and their targets have been identified by experimental or computational approaches, the majority of studies are focused on two model plant species: Arabidopsis thaliana and rice (Oryza sativa) [3], [5]. To further understand the function of plant miRNAs, more efforts should be made to include plant species with specific developmental features, which might contain miRNAs that are specific to these features [6].

miRNAs are characterized by their precursor stem-loop secondary structures and are conserved across species [7], [8]. The biogenesis of plant miRNAs is a complex multi-step enzymatic process [7], [9], [10]. miRNAs are initially transcribed by RNA polymerase II in the cell nucleus as long primary miRNAs that are cleaved into miRNA:miRNA* duplexes by the enzyme Dicer-like 1 (DCL1). Export of the duplexes into the cell cytoplasm is mediated by the protein HASTY. After methyl groups are added to the 3′ ends of the duplexes catalyzed by the protein HEN1, one strand of the duplexes is selectively incorporated into the RNA-induced silencing complex (RISC) to form the mature miRNAs, whereas the other strand, designated miRNA*, is typically degraded. Guided by miRNAs, the RISC recognizes the complementary sites on the target mRNAs and causes transcript cleavage [7], [11] or translational arrest [12], [13]. Recently, DCL4 has been shown to play a role in the biogenesis of a few miRNAs with long hairpin precursors [14].

Three major approaches are used for identifying miRNAs in plants: forward genetics, bioinformatic prediction and direct cloning and sequencing. Only a few miRNAs have been identified by forward genetic studies [12], [15] and predicting species-specific miRNAs by the bioinformatics method is difficult. Direct cloning and sequencing is the most effective method available for the discovery of plant miRNAs. Many groups have used this approach to clone and identify miRNAs in A. thaliana, O. sativa, cotton wood (Populus tricbocarpa), wheat (Triticum aestivum) and oilseed rape (Brassica napus) [16], [17], [18], [19], [20]. The development of high-throughput sequencing methods, such as the 454 Technology and the Solexa platform, has greatly improved this approach, which can identify low-abundance or tissue-specific miRNAs. However, there are some differences between these novel sequencing technologies. It is reported that the longest reads are obtained using the 454 Technology, whereas the Solexa platform can yield a higher number of reads [6], and is suitable for sequencing shorter reads (up to 35 bp) [21]. Because the miRNA sequences are only ∼21 nt in length, the Solexa platform appears to be preferred for miRNA discovery [22].

Peanut (also known as groundnut, Arachis hypogaea L.), an allotetraploid species (2n = 4x = 40; AABB), is one of the five most important oilseed crops cultivated worldwide [23]. Peanut seed contains ∼50% oil, of which ∼80% consists of oleic acids (36–67%) and linoleic acids (15–43%) [24]. The studies of the small RNAs in peanut have been reported [25], [26] but, compared with the number of miRNAs that have been identified in A. thaliana, O. sativa and P. trichocarpa, more miRNAs can be mined out from peanut. We describe the deep sequencing and analysis of small RNA transcriptomes from peanut using the high-throughput Solexa technology; 126 known miRNAs and 25 novel miRNAs were identified on the basis of either sequence similarity or the secondary structure of their precursors. We used quantitative real-time RT-PCR (qRT-PCR) to analyze the expression patterns of seven identified peanut miRNAs in different peanut tissues (root, stem, leaf, flower and seed) and different developmental stages of the seed. Furthermore, we made a deep analysis of peanut miRNA target functions using the GO database and KEGG pathway.

## Results

### Sequence analysis of short RNAs

We used high-throughput sequencing of small RNA libraries to identify low-abundance candidate miRNAs in peanut. In all, 25,686,617 reads were obtained from the Solexa sequencing machine for the small RNA library (mixed tissues of leaf, stem, root and seed of the cultivated peanut). After removing the adaptor/acceptor sequences, filtering the low-quality tags and cleaning up the contamination formed by the adaptor–adaptor ligation, 23,394,602 (91.08%) clean reads were obtained, representing 8,453,305 unique sequences. Among the total reads, 2,228,153 were found to be similar to miRNAs. The rest of the sequences were found to be other types of RNA, including noncoding RNA, tRNA, rRNA, snRNA or snoRNA. The numbers and proportions of different categories of small RNAs are given in Table 1.

**Table 1:** Distribution of small RNAs among different categories in peanut.

Category	Unique RNAs	Percent (%)	Total RNAs	Percent (%)
Total small RNAs	8,453,305	100	23,394,602	100
miRNA	22,767	0.27	2,228,153	9.52
rRNA	38,307	0.45	955,143	4.08
siRNA	264,353	3.13	2,069,895	8.85
snRNA	1,440	0.02	4,726	0.02
snoRNA	852	0.01	2,735	0.01
tRNA	8,213	0.10	742,543	3.17
Unannotated	8,117,373	96.03	17,391,407	74.34

**Figure 1:**
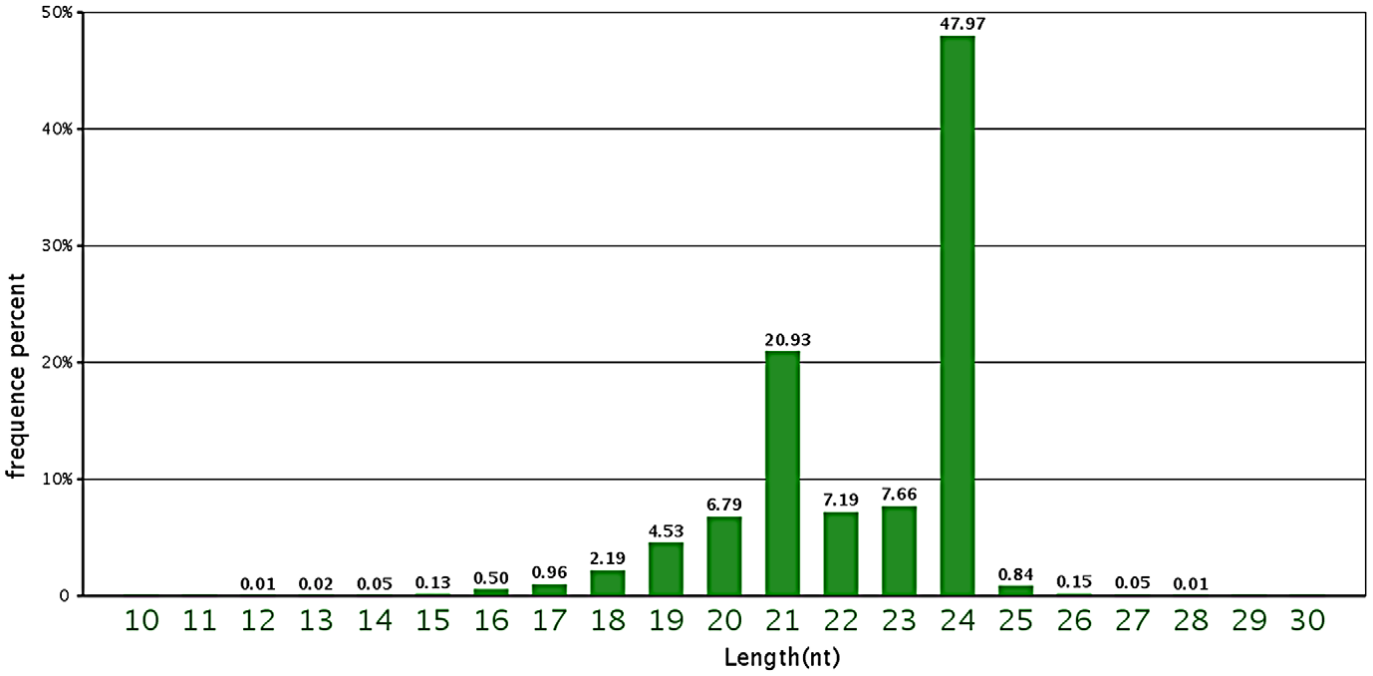
Length distribution and abundance of the sequences.

The composition of different categories of small RNAs often reflects the roles in a particular tissue or species and associated biogenetic machines. The majority of small RNAs from the libraries were 24 nt long (Figure 1) and accounted for 48% of the total sequence number, followed by 21 nt (20.9%), 23 nt (7.7%) and 22 nt (7.2%). This result was consistent with those reported for other plant species, including *A. thaliana*, *Medicago truncatula*, *O. sativa*, *Populus* spp. and *Citrus trifoliate*, where 24 nt sRNAs dominated the sRNA transcriptome [6], [14], [21], [22], [27], [28]. Such a high percentage of 24 nt small RNAs could reflect the complexity of the peanut genome because 24 nt siRNAs are known to be involved in heterochromatin modification, especially for a genome with a high content of repetitive sequences [29], [30].

### Identification of known miRNAs in peanut

There are 17,341 miRNAs from 133 species deposited in the miRBase database (Release 16.0, September 2010) and 2,846 miRNAs from 33 plant species belong to 632 different families. After removing repeat sequences, the 1,565 unique miRNA sequences were used as queries to search the potential miRNAs in peanut.

On the basis of sequence similarity, our analysis revealed that 126 known miRNAs were identified, which belong to 33 miRNA families with an average of about 4 miRNA members per family (Table S1). As expected, most of the miRNAs identified in peanut were highly conserved in diverse plant species [31], suggesting that the ancient regulatory pathways mediated by evolutionarily conserved miRNAs are present in legumes. We analyzed the miRNA members of known families and found significant divergence among them. The miR165 family was the largest identified, with 26 members that were distinguished by internal nucleotide differences. miR166 (17 members), miR167 (8 members) and miR169 (7 members) were the second, third and fourth miRNA families, respectively. Of the remaining 29 miRNA families, 13 contained 2–6 members, and 16 miRNA families were each represented by a single member.

The sequencing frequencies for miRNAs in the library can be used as an index for estimating the relative abundance of miRNAs. Solexa sequencing produced a large number of miRNA sequences, allowing us to determine the relative abundance of miRNAs in peanut; the frequencies of miRNA families varied from 1 (miR2914) to 1,330,176 (miR156), indicating that expression varies significantly among different miRNA families. Counting redundant miRNA reads revealed that 11 out of 33 conserved miRNA families were represented by more than 1,000 reads in the peanut dataset. The miR156 (1,330,176 reads), miR166 (215,652 reads) and miR167 (62,193 reads) families were the most frequent in the library. The majority of peanut miRNA families were sequenced less than 1,000 reads, and miR393, miR403, miR1507 and miR2914 were detected less than 10 reads. Sequence analysis indicated that the relative abundance of certain members within the miRNA families varied greatly in peanut, suggesting functional divergence within the family. For example, abundance of the miR156 family varied from 1 read (miR156f) to 474,415 reads (miR157) in the deep sequencing, similar to the case for some other miRNA families, such as miR166 (1–120,435 reads) and miR167 (2–57,060 reads). These results indicate that different members have clearly different expression levels in one miRNA family, probably because the expression is tissue- or developmental stage-specific.

### Novel miRNAs in peanut

For the identification of novel peanut miRNAs, we rely on peanut EST sequences as miRNA surrounding sequences in prediction, because details of the peanut genome sequence are limited. A total of 25 small RNAs met our criteria as established according to Allen *et al.* (2005) [32] and were considered putative novel peanut miRNAs (Table 2; Figure S1). Of these miRNAs, 4 candidates contained both miRNA and miRNA* sequences. We believe that the detection of miRNA*s is a strong clue, albeit not infallible, for the formation of precursor hairpin structures and added weight to the authenticity of the predicted candidates [27], [31]. However, the evolution and function of antisense miRNAs remains unclear. We propose that these miRNAs might differ from their sense partners by acting on different mRNA targets [33]. These novel candidates displayed a concentrated length distribution between 20 nt and 23 nt, with a peak at ∼21 nt. Precursors of these novel miRNAs had negative folding free energies ranging from −87.2 to −21.9 kcal mol^−1^, with an average of about −50.01 kcal mol^−1^ according to Mfold, which was similar to the free energy values of other plant miRNA precursors (−59.5 kcal mol^−1^ in *A. thaliana* and −71.0 kcal mol^−1^ in *O. sativa*). These values were much lower than the reported folding free energies of tRNA (−27.5 kcal mol^−1^) or rRNA (−33 kcal mol^−1^) [34]. The predicted hairpin structures for the precursors of these miRNAs required 75–343 nt, with a majority of the identified miRNA precursors (88%) requiring 75–188 nt, similar to what had been observed in *A. thaliana* and *O. sativa* [5].

**Table 2:** Novel miRNAs predicted from peanut.

Name	Count	Sequence	Length (nt)	EST no*	Precursor length (nt)	Folding energy (kcal mol^−1^)
miR1	1099	GAGAUCAGAGAUGCACACAUUU	22	C20R5_004_B09	109	−47.3
miR1*	81	AUGUGUGGGUUUCUGGUCUCCA	22	C20R5_004_B09	109	−47.3
miR2	235	GAGAUCAGAUCAUGUGGCAGU	21	C20R5_011_B12	92	−33.6
miR3	5716	UUCCAUACAUCAUCUAUCUAAC	22	C20R5_011_C07	116	−53.2
miR4	9	GGUUCUAGAUCGACGGUGGCA	21	C20R5_013_G06	76	−21.9
miR5	12	UUGGUAGCGGCGAAGCAGGA	20	C20R5_029_G05	100	−43.2
miR6	37	CAGGACCGGUGGAGUGUUAUGC	22	C20R5_054_H07	84	−48.4
miR7	1481	UUAUUGUCGGACUAAGGUGUCU	22	HS018_G07	114	−49.1
miR7*	1	ACACUUAGUCUUGCGAUAACU	21	HS018_G07	114	−49.1
miR8	157	GACUAAUCUGUCGCGGAUCU	20	HS019_H02	270	−87.2
miR9	5280	GCUCAAGAAAGCUGUGGGAGA	21	HS049_E06	143	−51.2
miR10	9	GGGUUCUAGAUCGACGGUGGC	21	HS273_C01	246	−68.4
miR11	5	UGUAUGGUGGAUGUAGGCAUU	21	HS274_G03	161	−56.3
miR12	16	AAAGAUAACAUAUAACUCUGC	21	TFL_001_D10	117	−21.9
miR13	8	CAUACGAGUUGUAAGAAGAAU	21	TFL_007_A09	107	−32.2
miR14	25	GAGGAAGAGGAGGAUGAAGGCC	22	TFR6_007_H02	152	−67.7
miR15	9	AGAGCUCUCAACUACCGGAGA	21	TFR7_024_G09	106	−47.4
miR16	1100	AGAGAUCAGAGAUGCACACAUU	22	ES717218	131	−50.3
miR16*	82	UGUGUGGGUUUCUGGUCUCCA	21	ES717218	131	−50.3
miR17	5	UUGUUUGCGAGUUGGGAUUUU	21	ES721379	139	−58.1
miR18	37	UCGCAGGACCGGUGGAGUGUUA	22	ES718804	123	−58.8
miR19	8	CAAGUGGUCUGCUACUAAAUU	21	GO257540	146	−32.2
miR20	13	UGAAUACCUCAUUCGGCCUCU	21	GO259494	343	−74.6
miR21	9	CACUGUUAUCAAUGGGUGUAUCU	23	GO266079	97	−23.12
miR22	5	GCUUGGAAGGAUGUUAGAGUA	21	GO268464	75	−24
miR23	21	UGACUGAAGUAGGAGGGAAAU	21	GO267334	141	−63.4
miR24	28	GGAGUGAAACUGAGAACACAAA	22	GO324297	151	−59.8
miR25	26	UAGGCUUAUGACCUCUUUCCA	21	GO325945	188	−65.1
miR25*	6	GAAAGAGUUUAUAAGCCUACU	21	GO325945	188	−65.1

We searched the nucleotide databases for homologs to determine whether these novel miRNAs are conserved among other plant species. This analysis indicated that miR2, miR4, miR9, miR10 and miR11 are conserved in other dicotyledonous plants (dicots), such as *Glycine max*, *Vicia faba*, *Vitis vinifera*, *M. truncatula* and *Trifolium pratense*. These findings indicate that these five miRNAs are conserved in dicots but not in rice or barley (*Hordeum vulgare*), suggesting that these are dicot-specific miRNAs.

The predicted novel miRNAs exhibited much lower expression levels, consistent to the notion that non-conserved miRNAs are often expressed at a lower level than conserved miRNAs. Only one member was identified in each novel miRNA family and 5 out of 25 novel miRNA families were sequenced more than 1,000 reads. The miR3 (5,716 reads) and miR9 (5,280 reads) families were the most frequent in the library. The majority of peanut miRNA families were sequenced less than 100 reads, and 9 families were detected less than 10 reads. The low abundance of novel miRNAs might suggest a specific role for these miRNAs under various growth conditions, in specific tissues, or during developmental stages. Whether these low-abundant miRNAs are expressed at higher levels in other tissues and organs, such as flowers, gynophores, or pods, or whether they are regulated by biotic or abiotic stress, remains to be investigated [25].

### Expression patterns of known and novel microRNAs identified in peanut

Knowledge of the expression patterns of miRNAs could provide clues about their functions [20]. To gain insight into the possible developmental stage- or tissue/organ-dependent roles of miRNAs in peanut, we examined the expression patterns of miRNAs in different tissues and in seed at different developmental stages.

**Figure 2:**
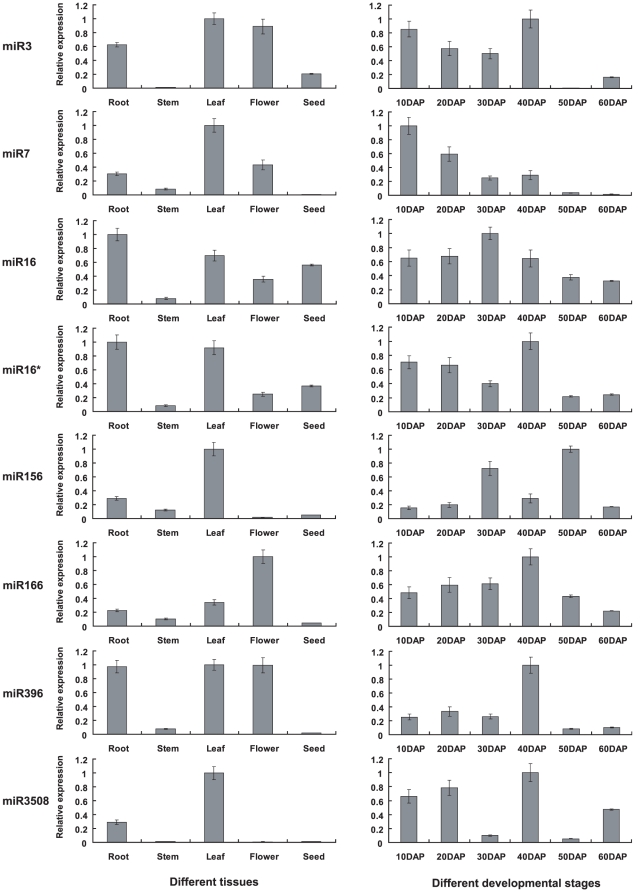
qRT-PCR validation and expression analysis of miRNAs in peanut.

The expression levels of 3 novel miRNAs (miR3, miR7, miR16 and miR16*) and 4 conserved miRNAs (miR156, miR166, miR396 and miR3508) were examined by stem-loop RT-PCR (Figure 2; Table 3). The expression patterns of miR3 and miR7 were similar: high expression in leaf, flower and root, and low expression in seed and stem. miR16 and miR16* had similar expression patterns: they were expressed abundantly in root and leaf, moderately in flower and seed and weakly in stem. This was reasonable because both mature miRNA and corresponding miRNA* were produced from the same precursors. Expression of miR156 was higher in leaf, root and stem, and lower in flower and seed. miR166 was expressed predominantly in flower followed by leaf and root, and weakly in seed. miR396 appeared to be highly expressed in leaf, root and flower but was detected only rarely in stem and seed. miR3508 expression appeared to be restricted to root and leaf. These observations suggested that most miRNAs in peanut are expressed preferentially in one or two tissues, and only some of them are highly expressed in multiple tissues examined.

**Table 3:** qRT-PCR-validated miRNAs and their sequences.

miRNA	Sequence
miR3	UUCCAUACAUCAUCUAUCUAAC
miR7	UUAUUGUCGGACUAAGGUGUCU
miR16	AGAGAUCAGAGAUGCACACAUU
miR16*	UGUGUGGGUUUCUGGUCUCCA
miR3508	UAGAGGGUCCCCAUGUUCUCA
miR156	UGACAGAAGAGAGUGAGCAC
miR166	UCGGACCAGGCUUCAUUCCCC
miR396	UUCCACAGCUUUCUUGAACUG

The expression patterns of the seven miRNAs across six developmental stages of seed are shown in Figure 2. The expression of miR3 was relatively high at the initial four stages, but showed a dramatic decrease in abundance during later stages; whereas miR7 reached a maximum level of expression at the initial stage and had a downward trend thereafter. miR16 and miR16* accumulated differently over six developmental stages, suggesting differential regulation of mature miRNAs and miRNA*s. miR166 showed gradual increases in abundance during earlier stages with highest expression at 40 days after pegging (DAP) and a gradual decrease thereafter. miR396 had the highest expression level at 40 DAP and much lower levels at other stages. The expressions of miR156 and miR3508 had no obvious pattern during seed development with a peak of RNA accumulation at 50 and 40 DAP, respectively.

### Prediction of miRNA targets in peanut

In order to better understand the biological functions of the newly identified as well as the known peanut miRNAs, we searched for putative target genes using the psRNATarget program with default parameters (http://plantgrn.noble.org/psRNATarget/). Under the strict criteria described in Materials and Methods, we found 381 potential targets with an average of 2.5 targets per miRNA molecule. The target genes were found for 32 known and 23 novel peanut miRNA families. Detailed annotation results of the best BLASTX hits against the NR protein database are given in Tables S2 and S3.

The putative target genes appeared to be involved in a broad range of biological processes and most of them were classified as transcription factors and functional proteins in plant metabolism and environmental stress response. These results are similar to those of earlier studies [25]. The predicted targets include homologs of known targets for conserved miRNAs and novel targets. As expected, many conserved miRNAs targeted transcription factors similar to those predicted in *A. thaliana* or soybean (*G. max*) [35], [36], such as those encoding the squamosa promoter-binding protein (SBP, ahy-miR156), the NAC domain protein (ahy-miR164), the auxin response factor (ARF, ahy-miR167), nuclear transcription factor Y (ahy-miR169), APETALA2 (AP2, ahy-miR172) and growth regulating factor (GRF, ahy-miR396), reinforcing the idea that conserved plant miRNAs are involved in essential biological processes. Other predicted targets included proteins such as transport inhibitor response 1 and auxin signaling F-box protein for ahy-miR393, resveratrol synthase and laccase for ahy-miR397, serine hydroxymethyltransferase for ahy-miR398, basic blue protein for ahy-miR408, polyphenol oxidase for ahy-miR3508 and transcripts that coded for unknown proteins. These observations suggested that the function of some well-conserved miRNAs had drifted during long periods of plant evolution. Although miRNAs are well conserved over long evolutionary timescales, some of their sequences have changed and display variations in a few nucleotide positions [37], which provides the chance for some miRNAs to base pair with other target mRNAs, exhibiting a species-specific regulatory pattern [38].

Unlike conserved miRNAs, the targets of novel peanut miRNAs were not enriched in transcription factors. Their target genes included those encoding the chlorophyll *a*/*b*-binding protein, heat shock protein, glycosyltransferase, diacylglycerol kinase family protein, aldo/keto reductase, formin-like protein, UDP-glucuronate 4-epimerase, cold-induced plasma membrane protein, aspartate carbamoyltransferase and nitrate transporter, implying that the corresponding novel miRNAs participate in some specific developmental processes in peanut. We predicted many genes with unknown function and hypothetical genes for miRNA targeting and careful analysis of these potential targets will contribute to our understanding of the role of miRNAs in legumes.

**Figure 3:**
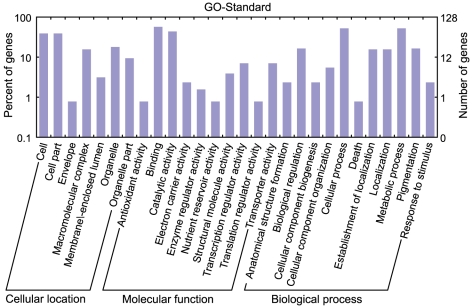
Gene categories and distribution of miRNA targets in peanut.

All targets regulated by peanut annotated miRNAs and novel miRNAs identified in this study were subjected to GO analysis to investigate gene ontology [39]. We found that 109 genes were involved in 68 different molecular functions, 87 genes took part in 52 biological processes and 46 genes participated in 21 cellular components (Figure 3; Table S4). Our GO biological process demonstrated that a total of 34 miRNA families could be involved in 52 different biological processes, such as response to stress, oxidation reduction, fatty acid biosynthesis, carbohydrate metabolism etc. Following GO analysis, we used KEGG to construct a pathway enrichment of predicted miRNA target genes (Table S4). Many metabolism networks were found to be involved, including plant–pathogen interaction, lipid metabolism, amino acid metabolism, carbohydrate metabolism, energy metabolism, nitrogen metabolism, signal transduction etc.

## Discussion

Although miRNAs have been studied extensively in the past several years, no systematic study has been reported for peanut, one of the most important oilseed crops cultivated worldwide. Recently, some miRNAs from peanut were identified by computational and direct cloning approaches [25], [26], but the identity and function of most peanut miRNAs are still largely unknown. Using high-throughput Solexa technology, we found evidence for the existence of 33 known miRNA families as well as 25 novel miRNA families in peanut. Five of these new miRNAs were found to be conserved in other dicots, including *G. max*, *V. faba* and *M. truncatula*, suggesting that they are dicot-specific. However, we did not find homologs of the remaining 20 miRNAs in other plants, and these might represent peanut-specific miRNAs. By deep sequencing of peanut small RNAs (6,009,541 reads), Zhao *et al.* (2010) identified 22 conserved miRNA families (miR156–miR894) and 14 novel miRNAs (miRn1–miRn14) [25]. Twenty-two of the conserved miRNA families and one of the novel miRNAs (miRn1 or miR3508) were identified as miRNAs in this study. Differences in tissue sampling and sequencing depth are likely to account for most of the differences of miRNAs identified between the two studies.

We used stem-loop RT-PCR to validate the predicted known and novel miRNAs and the results demonstrated that most of the tested miRNAs were expressed with tissue-, and/or growth stage-specific characteristics. Our qRT-PCR analysis validated the miRNA prediction in peanut and their preferential expression can provide important clues about where these miRNAs function. Our future work will focus on the demonstration of the role of these peanut miRNAs in control of peanut growth and development.

The expression analysis of ahy-miR156 revealed a tissue-specific expression pattern similar to that found in *A. thaliana*. ahy-miR156 showed higher expression levels in leaf, root and stem, and lower levels in seed and flower. In *A. thaliana*, miR156 was strongly expressed during seedling development and showed weak expression in mature tissues [40]. miR156 promoted juvenile development by repressing members of the SBP family of transcription factors. *O. sativa* miR156 showed an expression profile similar to those found in *A. thaliana* and peanut [41].

ahy-miR166 was expressed predominantly in flower followed by leaf and root, and weakly in seed, which was closely related to its functions. It has been proven that the miR166/165 group and its target genes regulate diverse aspects of plant development, including shoot apical and lateral meristem formation, leaf polarity, floral and root development, and vascular development [42], [43]. The *A. thaliana* miR166/165 group targets five members of the HD-ZIP III transcription factor genes and functions by cleaving target mRNAs through complementary base pairing [15], [44], [45]. Plants expressing 35S-MIR166g, which targeted members of the HD-ZIP transcription factor family, had radialized leaves, fasciated apical meristems and female sterility [15].

miR396, which is predicted to target GRF genes in *A. thaliana*, plays vital roles in plant growth, development and resistance to stress [46]. It has been reported that miR396-targeted AtGRF transcription factors are required for coordination of cell division and differentiation during leaf development in *A. thaliana* [47]. In addition, *A. thaliana* miR396 mediated the development of leaves and flowers in transgenic tobacco; over-expression of ath-miR396 in tobacco resulted in a small, narrow leaf phenotype and defects in the four whorls of floral organs [48]. Transgenic *O. sativa* and *A. thaliana* plants constitutively over-expressing osa-mir396c showed reduced salt and alkali stress tolerance compared to that of wild-type plants [49]. In this study, ahy-miR396 was preferentially expressed in leaf, root and flower, which is in good agreement with the research results discussed above.

Expression of miR3508, a legume-specific miRNA, appeared to be restricted to root and leaf. In peanut, it was predicted to target polyphenol oxidase (PPO) genes. PPO catalyzing the oxygen-dependent oxidation of phenols to quinones, which is ubiquitous among angiosperms, is localized on the thylakoids of chloroplasts and in vesicles or other bodies in non-green plastid types [50], [51]. This enzyme is responsible for the typical browning of plant extracts and damaged tissues and is assumed to be involved in plant defense against pests and pathogens [52]. Further study of the relationship between PPO and miR3508 should reveal the function of this pair in the regulation of peanut growth and development.

The expression of miR16*, the only miRNA* analyzed in this study, was closely related to that of mature miR16. It is reported that the relations between the accumulation abundance of mature miRNAs and miRNA*s are varied: some miRNAs and miRNA*s accumulate in the same tissue(s), whereas some accumulate in different tissues. In addition, for some miRNAs, the expression level of miRNA* was even higher than that of the corresponding miRNA [53]. Differential accumulation of miRNAs and miRNA*s could be due to the different activities of proteins involved directly in sRNA biogenesis [54].

A further step of target identification is necessary to assess and define a putative function for a miRNA in plant. Our analysis revealed that some of the predicted targets of conserved miRNAs in peanut had a conserved function with miRNA targets in *A. thaliana* and these miRNA target sequences were also highly conserved among a wide variety of plant species, as reported by Floyd and Bowman (2004) [55]. Consistent with earlier reports, some of these targets in peanut are plant-specific transcription factors, such as SBP, AP2, NAC, GRF and the ARF family. Moreover, some targets, especially for novel miRNAs, were distinct from *A. thaliana* and *O. sativa* genes, indicating that these targets might be involved in peanut-specific processes [20]. It will be interesting to identify the functions of these predicted target genes in peanut.

Cultivated peanuts are important oilseed crops worldwide, containing significant amounts of lipid and protein [23]. In this study, we searched for miRNAs that might play a function in regulating biological processes related to the biosynthesis of lipid and protein. Our results demonstrated that 4 miRNA families (miR156, miR159, miR171 and miR14) had a total of 4 targets, which were involved in amino acid metabolism, fatty acid metabolism and lipid metabolism. These results suggest that miRNAs might have an important role in lipid and protein accumulation in peanut.

In summary, we have identified large numbers of miRNAs from peanut, analyzed their expression and predicted the putative targets of these miRNAs. It will be very important to experimentally characterize these miRNAs and their downstream targets, as this will lead to better understanding of the function relationship and mechanism of miRNAs in the regulation network. Additionally, the deep sequencing approach to microRNA discovery suggests that a significant number of novel microRNAs remain to be discovered and characterized.

## Materials and Methods

### Ethics statement

No specific permits were required for the described field studies. No specific permissions were required for these locations and activities. The location is not privately-owned or protected in any way and the field studies did not involve endangered or protected species.

### Plant material

Peanuts (*A. hypogaea* L. cultivar Huayu19) were grown in a growth chamber with a 16 h light at 26°C/8 h dark at 22°C cycle. Leaf, stem, and root from 12 days old seedlings and immature peanut seeds were collected, immediately frozen in liquid nitrogen and stored at −80°C.

### Small RNA library development and sequencing

Mixed peanut tissues (leaf, stem, root and seed) were used. To identify as many tissue- or developmental stage-specific miRNAs as possible, we pooled the total RNAs from leaf, stem, root and seed samples in an equal fraction ratio. Total RNA was isolated using TRIzol® (Invitrogen, USA) then subjected to 15% (w/v) denaturing PAGE (polyacrylamide gel electrophoresis), after which the small RNA fragments of 18–28 nt were isolated from the gel and purified. Next, the small RNA molecules were ligated to a 5′ adaptor and a 3′ adaptor sequentially and then converted to DNA by RT-PCR. Finally, ∼20 µg products of RT-PCR were sequenced directly using a Solexa 1G genome analyzer according to the manufacturer's protocols (Beijing Genomics Institute, China) [56]. The sequenced short reads data have been deposited to the Short Read Archive section at NCBI under accession numbers SRA045592.

### Small RNA analysis

The raw data were first processed by the Fastx-toolkit pipeline to remove poor-quality reads and clip adapter sequences and sequences longer than 17 nt were used for further analysis. We searched against the Rfam database (http://www.sanger.ac.uk/software/Rfam) [57] and the GenBank noncoding RNA database (http://www.ncbi.nlm.nih.gov/) to remove these perfectly matched sequences, which might be noncoding RNAs (e.g. tRNA, rRNA, snRNA and snoRNA) or degradation fragments of mRNAs. In addition, the unique small RNA sequences were used to do a BLASTn search against the miRNA database, miRBase 16.0 [2], in order to identify known miRNAs in peanut. Only perfectly matched sequences were considered to be known miRNAs. All small RNA fragments and the identified orthologs of known miRNAs from miRBase were screened from expressed sequence tag (EST) sequences using the SOAP 2.0 program [58].

### Prediction of novel miRNA

Small RNA sequences were aligned to peanut ESTs to obtain precursor sequences in order to identify novel miRNAs in peanut. Contexts of perfectly matched hits were extracted as ±150 bp. The Mireap program developed by the Beijing Genome Institute (BGI) was used to analyze structural features of miRNA precursors to identify novel miRNA candidates. The resulting structures were retained as novel miRNA candidates only if they met the criteria described by Allen *et al.* (2005) [32]. The secondary structures of filtered pre-miRNA sequences were checked using Mfold [59]. In each case, only the structure with the lowest-energy was selected for manual inspection.

### Validation and expression analysis of peanut miRNAs using stem-loop RT-PCR

Samples of peanut leaf, stem, root, mature flower and seed from 10–60 DAP were collected from plants growing in the Shandong Peanut Research Institute. Each tissue type was collected in triplicate, immediately frozen in liquid nitrogen and stored at −80°C. Total RNA was extracted from each tissue sample using the mirVana miRNA isolation kit (Ambion, Austin, TX) according to the manufacturer's protocol. Total RNA was quantified and assessed for quality using a Nanodrop ND-1000 spectrophotometer (Nanodrop Technologies, Wilmington, DE). RNA samples were stored at −80°C until further analysis. Applied Biosystems TaqMan MicroRNA Assays were used to detect and quantify peanut miRNAs according to the manufacturer's protocol. Briefly, 1 µg of RNA from each tissue sample was used to generate a single-stranded miRNA cDNA by reverse transcription using the Applied Biosystems TaqMan MicroRNA Reverse Transcription Kit and miRNA-specific stem-loop primers provided with the kit. Next, the expression levels of seven peanut miRNAs were analyzed in five tissues and six seed developmental stages using qRT-PCR and miRNA-specific primers on a LightCycler 480 instrument system (Roche, Germany). 5.8S rRNA was used as the reference gene in qRT-PCR detection of peanut miRNAs. We analyzed changes in the expression levels of seven identified peanut miRNAs, which included 4 known miRNAs (miR156, miR166, miR396 and miR3508) and 3 peanut-specific miRNAs (miR3, miR7, miR16 and miR16*).

### Prediction of miRNA targets

The putative target sites of miRNA candidates were identified by aligning the miRNA sequences with the assembled ESTs of peanut using the psRNATarget program with default parameters (http://plantgrn.noble.org/psRNATarget/) [60].

All predicted target genes were evaluated by scoring, and the criteria used were: each G:U wobble pairing was assigned 0.5 point; each indel was assigned 2.0 points; and all other noncanonical Watson–Crick pairings were each assigned 1.0 point. The total score for an alignment was calculated on the basis of 20 nt; when the query was longer than 20 nt, scores for all possible consecutive 20 nt subsequences were computed, and the minimum score was considered to be the total score for the query–subject alignment. Because targets complementary to the miRNA 5′ end appear to be critical, mismatches other than G:U18 wobbles at positions 2–7 at the 5′ end were further penalized by 0.5 point in the final score [61]. Sequences were considered to be miRNA targets if the total score was less than 3.0 points [25].

### Analysis of GO and the KEGG pathway

In order to better understand miRNA target function and classification as well as the metabolic regulatory networks associated with peanut miRNAs and their targets, BLASTX was done with the target sequence and the NCBI database. All predicted target proteins with an *E* value of 1e-30 were identified by BLASTX searching against the Interpro and KEGG database and the best hits were used to validate the target gene function and metabolic pathway regulated by miRNAs. We obtained biological process, cellular component and molecular function of target genes the same as in the GO database by using the Interpro database.

## Supporting Information

Figure S1 **Examples of the predicated secondary structures of miRNAs in peanuts.** Red colored letter: mature miRNA sequence; blue colored letter: miRNA* sequence.
(DOC)

Table S1 **Conserved miRNAs from peanut.**
(DOC)

Table S2 **Identified targets of known miRNAs in peanut.**
(DOC)

Table S3 **Identified targets of new miRNAs in peanut.**
(DOC)

Table S4 **GO classification and KEGG pathway analysis of targets.**
(XLS)
