# Supplementary material for: Identification and Characterization of microRNAs from Peanut (Arachis hypogaea L.) by High-Throughput Sequencing
Source: PLoS One. 2011 Nov 16;6(11):e27530. doi: 10.1371/journal.pone.0027530 (PMC3217988; doi:10.1371/journal.pone.0027530)
Supplement: Figure S1 — Examples of the predicated secondary structures of miRNAs in peanuts. Red colored letter: mature miRNA sequence; blue colored letter: miRNA* sequence. (DOC) [file pone.0027530.s001.doc]

(1) miR1: GAGAUCAGAGAUGCACACAUUU

miR1* : AUGUGUGGGUUUCUGGUCUCCA

GU -|C A G U G U UGAG G

UGG G GU GAGAUCAGAGAU CACACAUU UACAUG CAU UGU GGUA C

ACC C CA CUCUGGUCUUUG GUGUGUAA GUGUAU GUG ACG UUAU A

AU U^A C G U G U ---- A

(2) miR2: GAGAUCAGAUCAUGUGGCAGU

CACU| A UN G UAA U CUCCUAU

AC AG GAAGCUGCCA CAUGAUUU CUU CCCU \

UG UC CUUUGACGGU GUACUAGA GAG GGGG G

----^ A CA - CUA U UUGUUUA

(3) miR3: UUCCAUACAUCAUCUAUCUAAC

G U U U - U GG U--| G

UGAG AC AUGUU GGUAGA GGUGUAUGGAAUGAAAAA GGGAAG CGU UUC G

AUUC UG UACAA CUAUCU CUACAUACCUUACUUUUU UCCUUC GUA GAG U

A - U U A C AA CUU^ U

(4) miR4: GGUUCUAGAUCGACGGUGGCA

UUUC -- C -| CGU UU C

AAGUCU UCACC UCG UCU AACUU GUUC \

UUCGGA GGUGG AGC AGA UUGGG CAAG C

GUUU AC C U^ UC- U- A

(5) miR5: UUGGUAGCGGCGAAGCAGGA

C UG CCA AA - A -| UC C

CGACUU CUUCUU UCGCC CU CCAAGG AG AGUAUAUU UC A

GUUGAA GAAGGA AGCGG GA GGUUCC UC UUAUAUGA AG A

- GA CGA C- U C U^ GA G

(6) miR6: CAGGACCGGUGGAGUGUUAUGC

AAUC| GACC UU CU AA

CUGUCGCAG GGUGGAGUG AUGC GCACGC G

GGCAGCGUC CCACCUCAC UAUG CGUGCG U

UUUC^ GA-- CU AC GA

(7) miR7: UUAUUGUCGGACUAAGGUGUCU

miR7*: ACACUUAGUCUUGCGAUAACU

A - U C AU-------| U

CUUUCUAGAGGACA CUUAGUCU GCGAUAA UUUAAUAUCAUU UAUAC A

GAAAGAUUUUCUGU GAAUCAGG UGUUAUU AAAUUAUAGUGA GUAUG A

G G C A CACAUUACU^ U

(8) miR8: GACUAAUCUGUCGCGGAUCU

CUUC CGCC C GA AG .-AA - .-UG UC C U C- .-AUCGUA| G

UAGU GGAUUCGC GCAGG GGUU UUUG GA GGUGGUUGC UCGAU GGAG AGC CG GGACCAA GUGGCC \

AUCA UCUAGGCG UGUCU UCAG GAGC CU UCACCAACG AGCUA CCUC UCG GU CCUGGUU CGCCGG U

UUAU AA-- C AA A- \ -- A \ -- C- - U CC \ ------^ U

(9) miR9: GCUCAAGAAAGCUGUGGGAGA

CUG C A .-AU| C

GUCAUG UUUUCCACAGCUUUCUUGA CUUCUUGU GUG A

CGGUAU AGAGGGUGUCGAAAGAACU GAAGAACG UAC U

UAA A C \ --^ C

(10) miR11: UGUAUGGUGGAUGUAGGCAUU

GAAAU AU- CU G .-AAUGAUA| CUGAAG UUG

GGAG UGCU UAUCCA UAUGCA UUAGCU CUCAUG \

CCUC ACGG GUAGGU GUAUGU AAUCGA GAGUAU A

CACAU GUU AU G \ -------^ AAA--- UAG

(11) miR13: CAUACGAGUUGUAAGAAGAAU

AUGACCAA| AC A UAA AAGU AACA AC

CAGU UCUUCU ACAACUCGU GA GAGAGG UGUUUUA \

GUUA AGAAGA UGUUGAGCA CU UUCUCC ACGGAGU U

CCCAUCAA^ -- A UA- ---- A--- CA

(12) miR14: GAGGAAGAGGAGGAUGAAGGCC

C C CU C C .-UA| AC A

UC UCUUCCUG CUUUCA CUUCCUCUUCC CC CUGU GC A

AG AGAAGGAC GGAAGU GGAGGAGAAGG GG GACG UG C

- A C- A A \ --^ GC U

(13) miR15: AGAGCUCUCAACUACCGGAGA

U| UG A C AU UAA GUGGU

CUU AAAUUUUUUCG UGGU GAGAGUUUUUCACG GGCC CAA A

GGG UUUAGAGAGGC AUCA CUCUCGAGAGGUGC CCGG GUU U

-^ GU C A -- UA- AAGAC

(14) miR16: AGAGAUCAGAGAUGCACACAUU

miR16*: UGUGUGGGUUUCUGGUCUCCA

------| UAAGUU C A G U G U UGAG G

AUUUCUGU GGG GU GAGAUCAGAGAU CACACAUU UACAUG CAU UGU GGUA C

UGAAGAUA CUC CA CUCUGGUCUUUG GUGUGUAA GUGUAU GUG ACG UUAU A

CAGGUA^ C----- A C G U G U ---- A

(15) miR17: UUGUUUGCGAGUUGGGAUUUU

AUCUACCU| C GG A GG U AAAA UGUG C

GGUUGUUUG GAGUUG AUUUU GAGG AAGAAAGGG GAA GGUUGU UAAAUU \

CCAACAAAC CUCAAC UAAAA CUCC UUCUUUUCC CUU UUAACA AUUUAA G

UCGUCC--^ U UU C UU - C--- UCCA A

(16) miR18: UCGCAGGACCGGUGGAGUGUUA

- CA | GACC UU CU AA

GA AUCCU--GUCGCAG GGUGGAGUG AUGC GCACGC G

CU UAGGG CAGCGUC CCACCUCAC UAUG CGUGCG U

C CC \ ^ GA-- CU AC GA

(17) miR19: CAAGUGGUCUGCUACUAAAUU

CAAG -- G C - .-UUCU| UACC

GCGACUC AAGU GUCUG UA CUAAA GAU A

CGUUGAG UUCA UAGAU AU GAUUU CUA C

GGUA AU - A C \ ----^ UCCU

(18) miR21: CACUGUUAUCAAUGGGUGUAUCU

C UGAGU - G UAUA -| GAAUCA

AUGUU GAUAC CU AUUGAUA UG GAUGUAUCU \

UAUAG CUAUG GG UAACUAU AC CUAUGUGGG U

A UCAUU U G UGUC U^ UAACUA

(19) miR22: GCUUGGAAGGAUGUUAGAGUA

UUUAUCUUG| U UU G G

CU UUCU UGU CCUUCCAAGUGUUU A

GA GAGA GUA GGAAGGUUCGUAAA C

UCUUUUUCA^ U UU - A

(20) miR23: UGACUGAAGUAGGAGGGAAAU

- U C GA U U GU --| GA UA

UUGUUUGUUU GA UGAAGUAGGAGGGAAAUA GGAGA GGA GAGAAAU CAAA UUUU UGUA \

AACAAACAAA CU ACUUUAUCCUCCCUUUAU UCUCU CCU CUCUUUA GUUU AAAG AUAU A

A C C A- C U AU UA^ AA UU

(21) miR24: GGAGUGAAACUGAGAACACAAA

UUAU| A AA A UUUUG ----- AUC U UUA

CUCAGAGG GUGA CUGAGAACACAA GUAAAUUG GAGUUUGGAA UGCCAUA ACA GCAU U

GAGUUUCC CACU GACUCUUGUGUU CAUUUAAU UUUAAGCUUU AUGGUAU UGU CGUA G

AACC^ C G- C ----- UAUAC A-- U UUG

(22) miR25: UAGGCUUAUGACCUCUUUCCA

miR25*: GAAAGAGUUUAUAAGCCUACU

- A UAU C C ---- UCGA--- .-U| UCUUU UAA

GUA CAA UAGGCUUAUGA CUCUUUC AUCGCAUUAU CAC AUUGUGAGC UAAUU UAUUGA U

CAU GUU AUCCGAAUAUU GAGAAAG UAGUGUAGUG GUG UGACACUCG GUUGA GUGGCU U

C A UUC U U CGCU UUGUGUG \ -^ UAAAU UCU

(23) miR3508: UAGAGGGUCCCCAUGUUCUCA

C UAUCUU CA C A C CUG- U .-CUCGUAA| U

UCC UGAGGACA GG G AUCCUCUAUUCU UCUU CAC GCCGC AUCAC A

AGG ACUCUUGU CC C UGGGAGAUAAGA AGAA GUG UGGUG UAGUG C

C UUNAAU AC - - - ACGA - \ -------^ U

Figure S1. Examples of the predicated secondary structures of miRNAs in peanuts. Red colored letter: mature miRNA sequence; blue colored letter: miRNA* sequence.
