# Supplementary material for: Identification and Characterization of microRNAs from Peanut (Arachis hypogaea L.) by High-Throughput Sequencing
Source: PLoS One. 2011 Nov 16;6(11):e27530. doi: 10.1371/journal.pone.0027530 (PMC3217988; doi:10.1371/journal.pone.0027530)
Supplement: Table S1 — Conserved miRNAs from peanut. (DOC) [file pone.0027530.s002.doc]

**Table S1.** Conserved miRNAs from peanut.

| **miRNA family** | **Name** | **Sequence (5’-3’)** | **Length (nt)** | **Reference miRNA** | **Conserved in** | | | | | | **Reads** |
| --- | --- | --- | --- | --- | --- | --- | --- | --- | --- | --- | --- |
|  |  |  |  |  | **ath** | **vvi** | **zma** | **osa** | **rco** | **gma** |  |
| 156 | miR156a | UGACAGAAGAGAGUGAGCAC | 20 | ath-miR156a | ++ | ++ | ++ | ++ | ++ | ++ | 461685 |
|  | miR156a | UGACAGAAGAGAGGGAGCAC | 20 | vvi-miR156a | + | ++ | + | + | + | + | 74 |
|  | miR156a | UGACAGAAGAGAGUGAGCGC | 20 | pts-miR156a | + | + | + | + | + | + | 56 |
|  | miR156b | UUGACAGAAGAUAGAGAGCAC | 21 | ahy-miR156b | ++ | ++ | + |  | ++ | ++ | 356563 |
|  | miR156b | CUGACAGAAGAUAGAGAGCAC | 21 | smo-miR156b | + | + |  |  | + | + | 94 |
|  | miR156c | UUGACAGAAGAGAGAGAGCAC | 21 | ahy-miR156c | + | + | ++ | + | + | ++ | 4611 |
|  | miR156c | UGUCAGAAGAGAGUGAGCAC | 20 | ghr-miR156c | + | + | + | + | + | + | 33 |
|  | miR156c | UUGACAGAAGAAAGAGAGCAC | 21 | smo-miR156c | + | + | + | + | + | + | 47 |
|  | miR156d | UGACAGAAGAGAGUGAGCACA | 21 | rco-miR156d | + | + | + | + | ++ | + | 18473 |
|  | miR156e | UGACAGAGGAGAGUGAGCAC | 20 | vvi-miR156e | + | ++ | + | + | + | + | 65 |
|  | miR156f | UUGACAGAAGAGAGAGAGCACA | 22 | gma-miR156f | + | + | + | + | + | ++ | 1 |
|  | miR156g | CGACAGAAGAGAGUGAGCAC | 20 | ath-miR156g | ++ | + | + | + | + | + | 144 |
|  | miR156g | UUGACAGAAGAUAGAGGGCAC | 21 | mtr-miR156g | + | + | + | + | + | + | 68 |
|  | miR156g | ACAGAAGAUAGAGAGCACAG | 20 | gma-miR156g | + | + |  |  | + | ++ | 1 |
|  | miR156h | UGACAGAAGAGAGAGAGCAU | 20 | vvi-miR156h | + | ++ | + | + | + | + | 2 |
|  | miR156h | UGACAGAAGAAAGAGAGCAC | 20 | ath-miR156h | ++ |  |  |  |  |  | 19 |
|  | miR156i | UGACAGAAGAUAGAGAGCAC | 20 | vvi-miR156i | ++ | ++ |  |  | ++ | ++ | 6752 |
|  | miR156j | UGACAGAAGAGAGAGAGCACA | 21 | zma-miR156j | + | + | ++ | ++ | ++ | ++ | 9 |
|  | miR156k | UGACAGAAGAGAGCGAGCAC | 20 | zma-miR156k | + | + | ++ | + | + | + | 166 |
|  | miR156o | UGACAGAAGAGAGUGAGCAU | 20 | osa-miR156o | + | + | + | ++ | + | + | 4946 |
|  | miR156p | UGACAGAAGAGAGUGAGCUC | 20 | osa-miR156p | + | + | + | ++ | + | + | 48 |
|  | miR156q | UGACAGAACAGAGUGAGCAC | 20 | osa-miR156q | + | + | + | ++ | + | + | 29 |
|  | miR156r | UGACAGAAGAGAGUGGGCAC | 20 | zma-miR156r | + | + | ++ | + | + | + | 63 |
|  | miR157a | UUGACAGAAGACAGAGAGCAC | 21 | nad-miR157a | + | + | + |  | + | + | 58 |
|  | miR157b | UUGACAGAAGAUAGAGAGCAU | 21 | sly-miR157b | + | + | + |  | + | + | 1754 |
|  | miR157 | UUGACAGAAGAGAGUGAGCAC | 21 | sbi-miR157 | + | + | + | + | + | + | 474415 |
| 159 | miR159a | UUUGGAUUGAAGGGAGCUCUA | 21 | ath-miR159a | ++ | ++ | + | + | ++ | ++ | 839 |
|  | miR159b | AUUGGAGUGAAGGGAGCUCCA | 21 | gma-miR159b | + | + | + | + | + | ++ | 12 |
|  | miR159b | UUUGGAUUGAAGGGAGCUCUU | 21 | ath-miR159b | ++ | + | + | + | + | + | 515 |
|  | miR159f | UUUGGAUUGAAGGGAGCUCUG | 21 | zma-miR159f | + | + | ++ | ++ | + | + | 13 |
|  | miR319a | UUGGACUGAAGGGAGCUCCCU | 21 | ath-miR319a | ++ | ++ | + | + | ++ | + | 34 |
|  | miR319b | CUUGGACUGAAGGGAGCUCCC | 21 | vvi-miR319b | + | ++ | + | + | + | + | 1 |
|  | miR319e | UUUGGACUGAAGGGAGCUCCU | 21 | vvi-miR319e | + | ++ | + | + | + | + | 12 |
|  | miR319g | UUGGACUGAAGGGAGCUCCCA | 21 | vvi-miR319g | + | ++ | + | + | + | + | 1 |
| 160 | miR160 | UGCCUGGCUCCCUGAAUGCCA | 21 | ahy-miR160 | + | ++ | + | ++ | ++ | + | 67 |
|  | miR160b | UGCCUGGCUCCCUGUAUGCCA | 21 | rco-miR160b | ++ | ++ | ++ | ++ | ++ | ++ | 492 |
|  | miR160 | GCCUGGCUCCCUGUAUGCCAU | 21 | csi-miR160 | + | + | + | + | + | + | 5 |
|  | miR160o | UGCCUGGCUCCCUGUAUGCCU | 21 | gma-miR160o | + | + | + | + | + | ++ | 3 |
| 162 | miR162a | UCGAUAAACCUCUGCAUCCAG | 21 | ath-miR162a | ++ | ++ | ++ | ++ | ++ | ++ | 1044 |
| 164 | miR164c | UGGAGAAGCAGGGCACGUGCA | 21 | rco-miR164c | ++ | ++ | ++ | ++ | ++ | ++ | 4341 |
|  | miR164c | UGGAGAAGCAGGGUACGUGCA | 21 | osa-miR164c | + | + | + | ++ | + | + | 4 |
|  | miR164d | UGGAGAAGCAGGGCACGUGCU | 21 | osa-miR164d | + | + | ++ | ++ | + | + | 50 |
| 166 | miR165a | UCGGACCAGGCUUCAUCCCCC | 21 | ath-miR165a | ++ | + | + | + | + | + | 49 |
|  | miR165b | UCGGACCAGGCUUCAUCCCC | 20 | ath-miR165b | ++ | + | + | + | + | + | 3 |
|  | miR166a | UCGGACCAGGCUUCAUUCCUG | 21 | vvi-miR166a | + | ++ | + | + | + | + | 33 |
|  | miR166a | UCGGACCAGGCUUCAUUCCCCC | 22 | csi-miR166a | + | ++ | + | + | + | + | 16 |
|  | miR166b | UCGGACCAGGCUUCAUUCCCGU | 22 | csi-miR166b | + | + | + | + | + | + | 142 |
|  | miR166b | UCGGACCAGGCUUCAUUCCUA | 21 | mtr-miR166b | + | + | + | + | + | + | 31 |
|  | miR166b | UCGGACCAGGCUUCAUUCCCUU | 22 | crt-miR166b | + | + | + | + | + | + | 6 |
|  | miR166c | UCGGACCAGGCUUCAUUCCCC | 21 | ath-miR166c | ++ | ++ | ++ | ++ | ++ | ++ | 120435 |
|  | miR166e | UCGAACCAGGCUUCAUUCCCC | 21 | osa-miR166e | + | + | + | ++ | + | + | 21 |
|  | miR166g | UCGGACCAGGCUUCAUUCCCCU | 22 | vvi-miR166g | + | ++ | + | + | + | + | 61 |
|  | miR166h | UCGGACCAGGCUUCAUUCCUC | 21 | osa-miR166h | + | + | ++ | ++ | + | ++ | 48745 |
|  | miR166i | UCGGAUCAGGCUUCAUUCCUC | 21 | osa-miR166i | + | + | + | ++ | + | + | 28 |
|  | miR166m | UCGGACCAGGCUUCAUUCCCU | 21 | osa-miR166m | + | + | + | ++ | + | ++ | 246 |
|  | miR166n | UCGGACCAGGCUUCAAUCCCU | 21 | zma-miR166n | + | + | ++ | ++ | + | + | 1 |
|  | miR166q | UCGGACCAGGCUUCAUUCCUU | 21 | ptc-miR166q | + | + | + | + | + | + | 48 |
|  | miR166q | UCGGACCAGGCUUCAUUCCCG | 21 | gma-miR166q | + | + | + | + | + | ++ | 45783 |
|  | miR166u | UCGGACCACGCUUCAUUCCCC | 21 | zma-miR166u | + | + | ++ | + | + | + | 4 |
| 167 | miR167a | UGAAGCUGCCAGCAUGAUCUAA | 22 | bna-miR167a | + | + | + | + | + | + | 15 |
|  | miR167b | UGAAGCUGACAGCAUGAUCUA | 21 | tae-miR167b | + | + | + | + | + | + | 2 |
|  | miR167b | UGAAGCUGCCAGCAUGAUCUA | 21 | rco-miR167b | ++ | ++ | ++ | ++ | ++ | ++ | 4328 |
|  | miR167c | UGAAGCUGCCAGCAUGAUCUC | 21 | vvi-miR167c | + | ++ | + | + | + | + | 56 |
|  | miR167d | UGAAGCUGCCAGCAUGAUCUGG | 22 | ath-miR167d | ++ | + | + | + | ++ | + | 498 |
|  | miR167e | UGAAGCUGCCAGCAUGAUCUG | 21 | osa-miR167e | ++ | ++ | ++ | ++ | ++ | ++ | 125 |
|  | miR167f | UGAAGCUGCCAGCAUGAUCUU | 21 | gma-miR167f | + | + | + | + | + | ++ | 57060 |
|  | miR167g | UGAAGCUGCCAGCAUGAUCUGA | 22 | gma-miR167g | + | + | + | + | + | ++ | 109 |
| 168 | miR168a | UCGCUUGGUGCAGGUCGGGAA | 21 | ath-miR168a | ++ | ++ | + | + | ++ | ++ | 15080 |
|  | miR168b | UCGCUUGGUGCAGAUCGGGAC | 21 | zma-miR168b | + | + | ++ | ++ | + | + | 18 |
|  | miR168 | UCGCUUGGUGCAGGUCGGGAC | 21 | stu-miR168 | + | + | + | + | + | + | 64 |
| 169 | miR169a | CAGCCAAGGAUGACUUGCCGA | 21 | zma-miR169a | ++ | ++ | ++ | ++ | + | ++ | 2 |
|  | miR169b | GCAGCCAAGGAUGACUUGCCG | 21 | gso-miR169b | + | + | + | + | + | + | 1 |
|  | miR169c | CAGCCAAGGAUGACUUGCCGG | 21 | ath-miR169c | ++ | ++ | ++ | ++ | ++ | ++ | 328 |
|  | miR169d | UGAGCCAAGGAUGACUUGCCG | 21 | gma-miR169d | ++ | + | + | + | ++ | ++ | 4 |
|  | miR169e | AGCCAAGGAUGACUUGCCGG | 20 | gma-miR169e | ++ | ++ | ++ | ++ | ++ | ++ | 12 |
|  | miR169h | UGAGCCAAGGAUGGCUUGCCG | 21 | vvi-miR169h | + | ++ | + | + | + | + | 3 |
|  | miR169l | AAGCCAAGGAUGACUUGCCGG | 21 | mtr-miR169l | + | + | + | + | + | + | 187 |
| 171 | miR171b | CGAGCCGAAUCAAUAUCACUC | 21 | gma-miR171b |  |  |  |  |  | ++ | 421 |
|  | miR171b | CGAGCCGAAUCAAUAUCACU | 20 | csi-miR171b |  |  |  |  |  | ++ | 9 |
|  | miR171b | ACGAGCCGAAUCAAUAUCACU | 21 | mtr-miR171b |  |  |  |  |  | + | 2 |
|  | miR171c | UUGAGCCGUGCCAAUAUCACG | 21 | ath-miR171c | ++ | ++ | + | + | ++ | + | 1 |
|  | miR171f | UUGAGCCGUGCCAAUAUCACA | 21 | zma-miR171f | + | + | ++ | + | + | + | 21 |
|  | miR171f | UUGAGCCGCGCCAAUAUCACU | 21 | vvi-miR171f | + | ++ | + | + | + | + | 11 |
| 172 | miR172a | AGAAUCUUGAUGAUGCUGCA | 20 | ath-miR172a | ++ | ++ | ++ | ++ | + | ++ | 27 |
|  | miR172b | AAAAUCUUGAUGAUGCUGCAU | 21 | gra-miR172b | + | + | + | + | + | + | 2 |
|  | miR172c | AGAAUCUUGAUGAUGCUGCAG | 21 | ath-miR172c | ++ | + | + | + | + | + | 147 |
|  | miR172d | AGAAUCUUGAUGAUGCUGCAU | 21 | osa-miR172d | ++ | + | ++ | + | + | ++ | 3712 |
|  | miR172e | GGAAUCUUGAUGAUGCUGCAU | 21 | ath-miR172e | ++ | + | ++ | + | ++ | + | 3 |
|  | miR172n | AGAAUCCUGAUGAUGCUGCAU | 21 | zma-miR172n | + | + | ++ | + | + | + | 1 |
| 390 | miR390b | AAGCUCAGGAGGGAUAGCGCC | 21 | rco-miR390b | ++ | ++ | ++ | ++ | ++ | ++ | 749 |
| 393 | miR393a | UCCAAAGGGAUCGCAUUGAUC | 21 | ath-miR393a | ++ | ++ | ++ | ++ | ++ | ++ | 2 |
|  | miR393b | UCCAAAGGGAUCGCAUUGAUCU | 22 | osa-miR393b | + | + | ++ | ++ | + | + | 2 |
| 394 | miR394 | UUGGCAUUCUGUCCACCUCC | 20 | ahy-miR394 | ++ | ++ | ++ | ++ |  |  | 167 |
| 396 | miR396a | UUCCACAGCUUUCUUGAACUA | 21 | vvi-miR396a | + | ++ | + | + | + | + | 1 |
|  | miR396b | UUCCAUAGCUUUCUUGAACUG | 21 | gcl-miR396b | + | + | + | + | + | + | 1 |
|  | miR396e | UUCCACAGCUUUCUUGAACUG | 21 | gma-miR396e | ++ | ++ | ++ | ++ | + | ++ | 1075 |
|  | miR396e | UUCCACAGCUUUCUUGAACUGU | 22 | gma-miR396e | + | + | + | + | + | ++ | 3 |
|  | miR396f | UUCCACAGCUUUCUUGAACUU | 21 | zma-miR396f | ++ | + | ++ | ++ | ++ | ++ | 623 |
|  | miR396g | UUCCACGGCUUUCUUGAACUU | 21 | ptc-miR396g | + | + | + | + | + | + | 2 |
| 397 | miR397a | UCAUUGAGUGCAGCGUUGAUG | 21 | ath-miR397a | ++ | ++ | + | ++ | ++ |  | 2091 |
|  | miR397b | UCAUUGAGUGCAGCGUUGAUGU | 22 | bna-miR397b | + | + | + | + | + |  | 29 |
|  | miR397b | UUAUUGAGUGCAGCGUUGAUG | 21 | osa-miR397b | + | + | + | ++ | + |  | 1 |
| 398 | miR398a | UGUGUUCUCAGGUCACCCCUU | 21 | vvi-miR398a | ++ | ++ | + | ++ | ++ | ++ | 1 |
|  | miR398b | UGUGUUCUCAGGUCGCCCCUG | 21 | rco-miR398b | + | ++ | + | ++ | ++ | + | 395 |
| 399 | miR399a | UGCCAAAGGAGAGUUGCCCUG | 21 | rco-miR399a | ++ | ++ | ++ | ++ | ++ | + | 13 |
|  | miR399a | UGCCAAAGGAGAUUUGCCCUG | 21 | ath-miR399a | ++ | + | + | + | + | + | 4 |
|  | miR399d | UGCCAAAGGAGAGCUGCCCUG | 21 | rco-miR399d | + | + | ++ | ++ | ++ | + | 4 |
|  | miR399h | UGCCAAAGGAGAGUUUCCCUG | 21 | ptc-miR399h | + | + | + | + | + | + | 1 |
|  | miR399i | CGCCAAAGGAGAGUUGCCCUG | 21 | vvi-miR399i | + | ++ | + | + | + |  | 40 |
| 403 | miR403b | UUAGAUUCACGCACAAACUCG | 21 | rco-miR403b | ++ | ++ |  |  | ++ |  | 6 |
| 408 | miR408 | AUGCACUGCCUCUUCCCUGGC | 21 | ahy-miR408 | ++ | ++ | + | + | + | + | 449 |
|  | miR408 | UGCACUGCCUCUUCCCUGGCUG | 22 | smo-miR408 | + | + | + | + | + | + | 2 |
| 528 | miR528b | UGGAAGGGGCAUGCAGAGGAG | 21 | zma-miR528b |  |  | ++ | ++ |  |  | 98 |
| 535 | miR535 | UGACAAUGAGAGAGAGCACAC | 21 | csi-miR535 | + | + | + | + | + | + | 33 |
|  | miR535c | UGACAACGAGAGAGAGCACGC | 21 | vvi-miR535c | + | ++ | + | ++ | ++ | + | 31 |
| 894 | miR894 | CGUUUCACGUCGGGUUCACC | 20 | ppt-miR894 |  |  |  |  |  |  | 51 |
| 1507 | miR1507 | CCUCGUUCCAUACAUCAUCUAG | 22 | mtr-miR1507 |  |  |  |  |  | ++ | 2 |
| 1511 | miR1511 | AACCAGGCUCUGAUACCAUG | 20 | gma-miR1511 | + | + |  | + |  | ++ | 36 |
| 1515 | miR1515 | UCAUUUUUGCGUGCAAUGAUCC | 22 | csi-miR1515 |  |  |  |  |  | + | 138 |
| 2018 | miR2018 | GCCCGUCUAGCUCAGUUGGU | 20 | tae-miR2018 |  |  |  |  |  |  | 15 |
| 2111 | miR2111a | UAAUCUGCAUCCUGAGGUUUA | 21 | ath-miR2111a | ++ | + |  |  |  |  | 26 |
| 2118 | miR2118 | UUGCCGAUUCCACCCAUUCCUA | 22 | gma-miR2118 |  | + |  |  |  | ++ | 157 |
| 2199 | miR2199 | UGAUACACUAGCACGGGUCAC | 21 | lja-miR2199 |  |  |  |  |  |  | 868 |
| 2910 | miR2910 | UAGUUGGUGGAGCGAUUUGUC | 21 | peu-miR2910 |  |  |  |  |  |  | 89 |
| 2914 | miR2914 | CAUGGUGGUGACGGGUGACGGAG | 23 | peu-miR2914 |  |  |  |  |  |  | 1 |
| 3508 | miR3508 | UAGAGGGUCCCCAUGUUCUCA | 21 | ahy-miR3508 |  |  |  |  |  |  | 22458 |

The abbreviations represent: ath, *Arabidopsis thaliana*; vvi, *Vitis vinifera*; zma, *Zea mays*; osa, *Oryza sativa*; rco, *Ricinus communis*; gma, *Glycine max*. The plus symbols indicate: ++, miRNA sequences of peanut were exactly identical to those in other species; +, miRNA sequences of peanut were conserved in other species but have variations in some nucleotide positions.
