# Supplementary material for: Identification and Characterization of microRNAs from Peanut (Arachis hypogaea L.) by High-Throughput Sequencing
Source: PLoS One. 2011 Nov 16;6(11):e27530. doi: 10.1371/journal.pone.0027530 (PMC3217988; doi:10.1371/journal.pone.0027530)
Supplement: Table S2 — Identified targets of known miRNAs in peanut. (DOC) [file pone.0027530.s003.doc]

**Table S2.** Identified targets of known miRNAs in peanut.

| **miRNA family** | **Target EST** | **Annotation** | **E-value** |
| --- | --- | --- | --- |
| miR156 | AHTC1003850 | hypothetical protein [*Ricinus communis*] | 7.00E-51 |
|  | AHTC1000296 | endo-1,3-beta-glucanase-like protein [*Pyrus pyrifolia*] | 8.00E-154 |
|  | AHTC1005919 | Ring/U-box domain-containing protein [*Arabidopsis thaliana*] | 3.00E-12 |
|  | AHTC1008113 | hypothetical protein [*Vitis vinifera*] | 6.04E-80 |
|  | AHTC1011110 | No hit found |  |
|  | AHTC1012203 | putative importin 9 [*Oryza granulata* ] | 2.00E-14 |
|  | AHTC1012670 | potassium channel beta [*Ricinus communis*] | 1.34E-72 |
|  | AHTC1016371 | No hit found |  |
|  | AHTC1016460 | hypothetical protein [*Vitis vinifera*] | 6.74E-28 |
|  | AHTC1019928 | hypothetical protein [*Arabidopsis lyrata*] | 5.21E-05 |
|  | AHTC1020639 | calmodulin-binding transcription activator | 1.38E-60 |
|  | AHTC1020982 | No hit found |  |
|  | AHTC1024353 | predicted protein [*Populus trichocarpa*] | 9.05E-05 |
|  | AHTC1029192 | No hit found |  |
|  | AHTC1029468 | hypothetical protein [*Ricinus communis*] | 1.68E-33 |
|  | AHTC1031460 | No hit found |  |
|  | AHTC1033143 | No hit found |  |
|  | AHTC1033793 | cationic amino acid transporter [*Populus trichocarpa*] | 2.10E-63 |
|  | AHTC1034362 | predicted protein [*Populus trichocarpa*] | 2.06E-07 |
|  | AHTC1034398 | ring finger protein [*Ricinus communis*] | 4.70E-22 |
|  | AHTC1034659 | eukaryotic translation initiation factor 2c [*Ricinus communis*] | 1.88E-58 |
|  | Contig10139 | hypothetical protein [*Vitis vinifera*] | 4.13E-41 |
|  | Contig102 | No hit found |  |
|  | Contig104 | No hit found |  |
|  | Contig10690 | No hit found |  |
|  | Contig120 | L-aspartate oxidase [*Phaseolus vulgaris*] | 2.30E-28 |
|  | Contig126 | No hit found |  |
|  | Contig127 | short-chain dehydrogenase reductase family protein | 3.40E-55 |
|  | Contig12819 | hypothetical protein [*Ricinus communis*] | 9.52E-57 |
|  | Contig129 | elongation factor 1-alpha | 1.34E-05 |
|  | Contig15800 | No hit found |  |
|  | Contig15803 | predicted protein [*Populus trichocarpa*] | 2.27E-12 |
|  | Contig16626 | squamosa promoter-binding protein [*Arabidopsis thaliana*] | 8.00E-37 |
|  | Contig17238 | unknown [*Arachis hypogaea*] | 1.05E-60 |
|  | Contig20181 | hypothetical protein [*Vitis vinifera*] | 2.00E-37 |
|  | Contig21951 | uncharacterized protein [*Glycine max*] | 7.00E-10 |
|  | Contig3535 | unknown [*Glycine max*] | 2.65E-19 |
|  | Contig42 | unknown [*Glycine max*] | 1.56E-114 |
|  | Contig43 | calcium-dependent lipid-binding domain-containing protein | 2.00E-69 |
|  | Contig44 | hypothetical protein [*Vitis vinifera*] | 9.01E-32 |
|  | Contig5833 | 26s proteasome regulatory subunit n3 | 5.52E-27 |
|  | Contig64 | No hit found |  |
|  | Contig69 | zinc ring-type | 2.92E-09 |
|  | Contig73 | protein kinase-domain containing protein [*Arabidopsis thaliana*] | 1.15E-58 |
|  | Contig77 | hypothetical protein [*Vitis vinifera*] | 4.30E-12 |
|  | Contig81 | serine racemase | 3.71E-11 |
|  | Contig90 | No hit found |  |
|  | Contig9345 | lactosylceramide 4-alpha-galactosyltransferase [*Ricinus communis*] | 3.02E-37 |
|  | Contig9597 | SH3 domain-containing protein [*Arabidopsis thaliana*] | 2.69E-19 |
|  | Contig96 | hypothetical protein [*Vitis vinifera*] | 1.16E-29 |
|  | Contig98 | No hit found |  |
|  | GW936404 | No hit found |  |
|  | GW939428 | No hit found |  |
|  | GW940294 | No hit found |  |
|  | GW953076 | unknown [*Glycine max*] | 1.09E-26 |
|  | GW971649 | No hit found |  |
|  | GW976292 | uncharacterized protein [*Glycine max*] | 7.00E-57 |
|  | GW977770 | sterol 24-C methyltransferase [*Glycine max*] | 2.00E-06 |
|  | GW979154 | No hit found |  |
|  | HS064_D06 | GH3 family protein [*Populus trichocarpa*] | 3.59E-45 |
|  | HS071_F08 | predicted protein [*Populus trichocarpa*] | 9.86E-10 |
|  | TFL_065_B03 | ribulose bisphosphate carboxylase small chain, chloroplastic [*Malus* sp.] | 1.00E-12 |
| miR159 | AHTC1000547 | ribulose bisphosphate carboxylase small chain [*Glycine max*] | 1.00E-73 |
|  | AHTC1002540 | unknown [*Medicago truncatula*] | 1.42E-116 |
|  | AHTC1011321 | unknown [*Glycine max*] | 1.27E-65 |
|  | AHTC1012025 | glycosyltransferase [*Medicago truncatula*] | 1.46E-75 |
|  | AHTC1013508 | galactokinase[*Ricinus communis*] | 6.00E-81 |
|  | Contig68 | unknown [*Glycine max*] | 2.22E-26 |
|  | Contig3238 | predicted protein [*Populus trichocarpa*] | 3.04E-16 |
|  | Contig5746 | glycoside hydrolase family 1 protein [*Leucaena leucocephala*] | 3.06E-94 |
|  | Contig86 | unknown [*Medicago truncatula*] | 4.04E-24 |
|  | GW948320 | No hit found |  |
|  | GW950762 | endo-1,4-beta-glucanase, putative [*Ricinus communis*] | 7.49E-88 |
|  | GW973747 | No hit found |  |
| miR160 | Contig91 | unknown [*Glycine max*] | 4.37E-24 |
|  | Contig118 | BEL1-like homeodomain transcription factor [*Trifolium pratense*] | 1.48E-60 |
|  | Contig97 | predicted protein [*Populus trichocarpa*] | 3.22E-81 |
| miR164 | AHTC1007323 | NAC domain protein [*Glycine max*] | 2.00E-133 |
|  | AHTC1022619 | NAC1 [*Medicago truncatula*] | 1.06E-111 |
|  | AHTC1029847 | unknown [*Glycine max*] | 1.21E-24 |
|  | AHTC1033856 | No hit found |  |
|  | Contig123 | unknown [*Medicago truncatula*] | 2.79E-47 |
|  | Contig4766 | unknown [*Glycine max*] | 1.36E-107 |
|  | Contig54 | NAC domain-containing protein [*Ricinus communis*] | 7.62E-31 |
|  | Contig6658 | NAC domain protein [*Glycine max*] | 3.00E-27 |
|  | Contig859 | NAC domain-containing protein [*Ricinus communis*] | 2.00E-34 |
| miR166 | AHTC1018155 | AMP-dependent synthetase and ligase | 4.08E-29 |
|  | AHTC1034734 | plastidic glucose transporter 4 | 2.54E-44 |
|  | Contig105 | ubiquitin carboxyl-terminal hydrolase 6 | 1.25E-47 |
|  | Contig52 | pentatricopeptide repeat-containing protein [*Ricinus communis*] | 2.00E-17 |
|  | Contig55 | pentatricopeptide repeat-containing protein [*Ricinus communis*] | 1.00E-79 |
|  | Contig85 | small subunit ribosomal protein [*Arabidopsis thaliana*] | 5.59E-10 |
|  | Contig89 | unknown [*Medicago truncatula*] | 8.81E-15 |
| miR167 | AHTC1009505 | one-helix protein [*Camellia sinensis*] | 8.26E-33 |
|  | AHTC1021829 | hypothetical protein [*Sorghum bicolor*] | 3.3 |
|  | Contig14589 | No hit found |  |
|  | Contig5589 | similar to auxin response factor 8 [*Vitis vinifera*] | 3.00E-95 |
|  | Contig79 | No hit found |  |
|  | GW971771 | sphingomyelin synthetase family protein | 1.24E-22 |
| miR168 | AHTC1034659 | eukaryotic translation initiation factor 2c [*Ricinus communis*] | 1.00E-59 |
|  | Contig56 | coatomer alpha subunit-like protein [*Lotus japonicus*] | 3.00E-21 |
|  | Contig87 | endomembrane family protein 70 [*Arabidopsis thaliana*] | 1.93E-56 |
| miR169 | AHTC1002398 | nuclear transcription factor Y subunit A-1 [*Ricinus communis*] | 1.00E-113 |
|  | AHTC1002707 | nuclear transcription factor Y subunit A-3 [*Ricinus communis*] | 3.00E-64 |
|  | AHTC1004127 | histone H2A [*Zea mays*] | 6.00E-33 |
|  | AHTC1008309 | nuclear transcription factor Y subunit A-3 [*Ricinus communis*] | 1.00E-45 |
|  | AHTC1013732 | No hit found |  |
|  | AHTC1020885 | calcium-binding EF-hand domain-containing protein [A*rabidopsis thaliana*] | 4.00E-24 |
|  | AHTC1028688 | predicted protein [*Populus trichocarpa*] | 8.71E-18 |
|  | AHTC1034734 | plastidic glucose transporter 4 | 2.54E-44 |
|  | C20R5_034_D12 | nuclear transcription factor Y subunit A-3, putative [*Ricinus communis*] | 1.00E-33 |
|  | Contig104 | No hit found |  |
|  | Contig106 | nuclear transcription factor y subunit a-1 | 1.71E-27 |
|  | Contig110 | nuclear transcription factor y subunit a-10 | 1.55E-70 |
|  | Contig114 | nuclear transcription factor y subunit a | 4.07E-80 |
|  | Contig117 | unknown [*Glycine max*] | 5.43E-09 |
|  | Contig15371 | multidrug resistance-associated protein, abc-transoprter [*Ricinus communis*] | 2.24E-107 |
|  | Contig18350 | No hit found |  |
|  | Contig20256 | uncharacterized protein [*Ricinus communis*] | 3.00E-10 |
|  | Contig24557 | No hit found |  |
|  | Contig46 | histone 2 | 2.16E-42 |
|  | Contig55 | hypothetical protein [*Vitis vinifera*] | 2.67E-67 |
|  | Contig60 | nuclear transcription factor y subunit a | 2.76E-69 |
|  | Contig71 | No hit found |  |
|  | ES718171 | nuclear transcription factor y subunit a-3 | 7.26E-47 |
|  | GW943466 | unknown [*Glycine max*] | 1.78E-10 |
| miR171 | AHTC1003885 | zinc ion transmembrane transporter [*Medicago sativa*] | 3.00E-87 |
|  | AHTC1006564 | 1-acyl-sn-glycerol-3-phosphate acyltransferase [*Prunus mume*] | 4.00E-86 |
|  | AHTC1021050 | pentatricopeptide repeat-containing protein [*Arabidopsis lyrata*] | 8.00E-55 |
|  | AHTC1031881 | hypothetical protein [*Vitis vinifera*] | 1.79E-56 |
|  | Contig14803 | pentatricopeptide repeat-containing protein [*Ricinus communis*] | 0.017 |
|  | Contig45 | unknown [*Glycine max*] | 2.10E-15 |
|  | Contig50 | 1-acyl-sn-glycerol-3-phosphate acyltransferase [*Prunus mume*] | 1.13E-18 |
|  | Contig5476 | zinc ion transmembrane transporter [*Medicago truncatula*] | 9.00E-15 |
|  | Contig5487 | 1-acyl-sn-glycerol-3-phosphate acyltransferase [*Prunus mume*] | 9.00E-21 |
|  | Contig78 | unknown [*Populus trichocarpa*] | 3.34E-05 |
|  | Contig82 | GRAS family transcription factor [*Populus trichocarpa*] | 6.00E-33 |
|  | GW933766 | oligopeptide transporter opt family [*Medicago truncatula*] | 3.64E-83 |
|  | GW939948 | No hit found |  |
| miR172 | AHTC1000914 | chaperone protein dnaJ [*Ricinus communis*] | 5.00E-45 |
|  | AHTC1014208 | unknown [*Glycine max*] | 7.88E-10 |
|  | AHTC1018174 | DNA binding protein [*Ricinus communis*] | 1.27E-53 |
|  | AHTC1028964 | unknown [*Glycine max*] | 3.67E-08 |
|  | AHTC1034123 | AP2 (APETALA 2) domain class transcription factor [*Malus x domestica*] | 1.66E-04 |
|  | Contig109 | predicted protein [*Populus trichocarpa*] | 1.93E-28 |
|  | Contig19067 | chaperone protein dnaJ [*Ricinus communis*] | 3.00E-13 |
|  | Contig3621 | No hit found |  |
|  | Contig37 | chaperone protein dnaJ [*Ricinus communis*] | 7.21E-12 |
|  | Contig62 | dihydrodipicolinate reductase [*Ricinus communis*] | 1.92E-13 |
|  | Contig63 | ethylene receptor | 8.71E-10 |
|  | Contig88 | No hit found |  |
|  | Contig91 | unknown [*Glycine max*] | 4.37E-24 |
| miR390 | Contig113 | protein kinase [*Glycine max*] | 2.54E-32 |
|  | Contig61 | No hit found |  |
|  | GW942061 | No hit found |  |
| miR393 | AHTC1002936 | protein auxin signaling F-box [*Arabidopsis thaliana*] | 0.0 |
|  | AHTC1007495 | unnamed protein product [*Vitis vinifera*] | 2.41E-130 |
|  | AHTC1008083 | transport inhibitor response 1 [*Dimocarpus longan*] | 7.00E-58 |
|  | Contig107 | n-acetylglucosaminyltransferase-like protein | 1.27E-32 |
|  | Contig19826 | transport inhibitor response 1 protein [*Ricinus communis*] | 3.00E-53 |
|  | Contig22836 | hypothetical protein [*Ricinus communis*] | 1.66E-30 |
|  | Contig41 | hypothetical protein [*Vitis vinifera*] | 3.33E-48 |
|  | Contig7568 | unknown [*Glycine max*] | 4.14E-11 |
|  | Contig8466 | predicted protein [*Populus trichocarpa*] | 1.03E-09 |
| miR394 | AHTC1026408 | hypothetical protein [*Sporosarcina newyorkensis*] | 0.42 |
|  | AHTC1035541 | gag-pol polyprotein [*Phaseolus vulgaris*] | 3.83E-12 |
|  | Contig122 | No hit found |  |
|  | Contig51 | predicted protein [*Populus trichocarpa*] | 1.73E-11 |
|  | GO263892 | predicted protein [*Populus trichocarpa*] | 2.00E-59 |
|  | GW982188 | unknown [*Medicago truncatula*] | 1.85E-25 |
| miR396 | AHTC1001528 | uncharacterized protein [*Medicago truncatula*] | 2.00E-133 |
|  | AHTC1005991 | growth-regulating factor 1 [*Oryza sativa*] | 4.00E-67 |
|  | AHTC1006636 | growth-regulating factor 5 | 1.24E-46 |
|  | AHTC1006736 | growth-regulating factor 5 | 7.25E-55 |
|  | AHTC1007044 | growth-regulating factor 12 [*Oryza sativa*] | 4.00E-24 |
|  | AHTC1008460 | No hit found |  |
|  | AHTC1010613 | uncharacterized protein [*Ricinus communis*] | 2.00E-35 |
|  | AHTC1012168 | predicted protein [*Populus trichocarpa*] | 1.00E-56 |
|  | AHTC1012558 | uncharacterized protein [*Arabidopsis thaliana*] | 1.00E-36 |
|  | AHTC1013123 | Ring-H2 finger protein [*Ricinus communis*] | 1.00E-46 |
|  | AHTC1016221 | growth-regulating factor 5 | 9.80E-44 |
|  | AHTC1016568 | uncharacterized protein [*Vitis vinifera*] | 7.00E-71 |
|  | AHTC1030474 | phosphoglycerate bisphosphoglycerate mutase family protein | 5.43E-48 |
|  | AHTC1031199 | eukaryotic translation initiation factor sui1 family protein | 3.12E-58 |
|  | Contig101 | hypothetical protein [*Sorghum bicolor*] | 3.88E-05 |
|  | Contig115 | hypersensitive-induced response protein 2 | 2.19E-132 |
|  | Contig125 | No hit found |  |
|  | Contig21390 | growth-regulating factor [*Zea mays*] | 2.00E-09 |
|  | Contig2270 | predicted protein [*Populus trichocarpa*] | 3.47E-72 |
|  | Contig24692 | No hit found |  |
|  | Contig34 | en spm-like transposon protein | 1.34E-13 |
|  | Contig53 | No hit found |  |
|  | Contig57 | phosphoribosylanthranilate isomerase | 5.44E-15 |
|  | Contig6241 | hypothetical protein [*Ricinus communis*] | 2.99E-08 |
|  | Contig70 | hypothetical protein [*Vitis vinifera*] | 7.22E-34 |
|  | Contig80 | hypothetical protein [*Vitis vinifera*] | 3.98E-16 |
|  | Contig8937 | Myrcene synthase, chloroplastic [*Quercus ilex*] | 2.00E-37 |
|  | GW939963 | No hit found |  |
|  | GW970961 | uncharacterized protein [*Arabidopsis thaliana*] | 2.00E-32 |
| miR397 | AHTC1000766 | resveratrol synthase [*Arachis hypogaea*] | 0.0 |
|  | AHTC1000768 | stilbene synthase 3 [*Arachis hypogaea*] | 0.0 |
|  | AHTC1009669 | heat shock protein 91 [*Arabidopsis thaliana*] | 3.00E-80 |
|  | AHTC1010790 | heat shock protein 70 [*Arabidopsis thaliana*] | 4.00E-92 |
|  | AHTC1011341 | potassium channel beta [*Ricinus communis*] | 8.00E-74 |
|  | AHTC1014167 | uncharacterized protein [*Glycine max*] | 3.00E-55 |
|  | AHTC1016936 | laccase [*Solanum lycopersicum*] | 6.00E-45 |
|  | AHTC1019858 | laccase [*Ricinus communis*] | 9.00E-104 |
|  | AHTC1021401 | putative dihydrolipoamide S-acetyltransferase [*Arabidopsis thaliana*] | 3.00E-06 |
|  | AHTC1033783 | disease resistance-responsive (dirigent-like protein) family protein | 1.97E-17 |
|  | AHTC1034127 | No hit found |  |
|  | AHTC1036625 | stilbene synthase [*Arachis hypogaea*] | 6.00E-110 |
|  | Contig121 | No hit found |  |
|  | Contig22711 | heat-shock protein [*Arabidopsis thaliana*] | 4.00E-51 |
|  | Contig23876 | laccase [*Ricinus communis*] | 1.00E-14 |
|  | Contig300 | heat shock protein [*Arabidopsis thaliana*] | 7.00E-45 |
|  | Contig35 | resveratrol synthase [*Arachis hypogaea*] | 0.0 |
|  | Contig47 | casein kinase beta polypeptide | 1.62E-108 |
|  | Contig67 | heat shock protein 70 | 1.36E-45 |
|  | Contig74 | putative copper ion-binding laccase [*Pisum sativum*] | 4.63E-09 |
|  | Contig76 | diphenol oxidase 10 | 3.66E-13 |
|  | Contig8894 | molybdopterin synthase sulphurylase | 2.73E-65 |
|  | Contig92 | No hit found |  |
|  | GW933073 | uncharacterized protein [*Arabidopsis thaliana*] | 8.65E-52 |
|  | GW947367 | unnamed protein product [*Vitis vinifera*] | 7.85E-09 |
|  | HS076_F06 | unknown [*Medicago truncatula*] | 7.14E-65 |
|  | TFL_039_C01 | threonine and homoserine efflux system | 3.35E-99 |
| miR398 | AHTC1011247 | unknown [*Glycine max*] | 5.41E-09 |
|  | AHTC1030590 | protein kinase [*Glycine max*] | 3.00E-69 |
|  | Contig100 | protein kinase [*Glycine max*] | 1.48E-52 |
|  | Contig26440 | unknown [*Glycine max*] | 5.21E-42 |
|  | Contig36 | serine hydroxymethyltransferase [*Glycine max*] | 0.0 |
|  | Contig99 | No hit found |  |
| miR399 | AHTC1031117 | No hit found |  |
|  | AHTC1010943 | aldose 1-epimerase [*Ricinus communis*] | 7.82E-69 |
|  | AHTC1036342 | predicted protein [*Populus trichocarpa*] | 1.32E-48 |
|  | Contig116 | No hit found |  |
|  | Contig142 | chaperone protein dnaJ, putative [*Ricinus communis*] | 9.52E-71 |
|  | Contig1848 | glycoside hydrolase family 1 protein [*Leucaena leucocephala*] | 8.74E-74 |
|  | Contig38 | glutathione s-transferase | 6.18E-70 |
|  | Contig9238 | No hit found |  |
| miR403 | AHTC1029755 | predicted protein [*Populus trichocarpa*] | 2.43E-18 |
|  | Contig3969 | hydroxyphenylpyruvate reductase | 8.56E-55 |
|  | GW935510 | cytochrome P450 [*Ricinus communis*] | 2.15E-39 |
| miR408 | AHTC1004566 | blue copper protein | 2.40E-37 |
|  | AHTC1009666 | basic blue copper protein [*Cicer arietinum*] | 9.00E-50 |
|  | AHTC1030408 | No hit found |  |
|  | Contig119 | unknown [*Glycine max*] | 8.88E-13 |
|  | Contig3998 | No hit found |  |
|  | Contig65 | No hit found |  |
|  | Contig66 | basic blue copper protein | 2.16E-11 |
|  | Contig72 | histone h1/h5 [*Ricinus communis*] | 8.39E-04 |
|  | Contig8198 | basic blue protein [*Cucumis sativus*] | 3.00E-10 |
|  | Contig84 | atidd14-domain 14 [*Arabidopsis lyrata*] | 3.17E-14 |
| miR528 | AHTC1004572 | putative DEAD-box protein abstrakt [*Trifolium pretense*] | 9.00E-51 |
|  | AHTC1020931 | cleavage and polyadenylation specificity factor [*Ricinus communis*] | 6.00E-53 |
|  | AHTC1026854 | glutamine synthetase [*Glycine max*] | 5.00E-68 |
|  | AHTC1032213 | unnamed protein product *[Vitis vinifera*] | 3.37E-57 |
|  | AHTC1034857 | unnamed protein product [*Vitis vinifera*] | 9.50E-07 |
|  | Contig108 | unknown [*Glycine max*] | 1.36E-12 |
|  | Contig111 | leucine-rich repeat family protein / extensin family protein [*Glycine max*] | 9.87E-93 |
|  | Contig25734 | No hit found |  |
|  | Contig4018 | unknown [*Glycine max*] | 3.86E-103 |
|  | Contig49 | unknown [*Glycine max*] | 8.80E-112 |
|  | Contig6025 | phosphatidylinositol 4-kinase [*Arabidopsis thaliana*] | 4.00E-44 |
|  | GO257033 | unknown [*Glycine max*] | 1.72E-34 |
| miR535 | Contig112 | glycosyltransferase [*Ricinus communis*] | 7.87E-57 |
|  | Contig4215 | hypothetical protein [*Vitis vinifera*] | 4.90E-09 |
|  | Contig9098 | dna-3-methyladenine glycosylase i | 1.08E-31 |
|  | GW948372 | crt (chloroquine-resistance transporter)-like transporter 1 | 3.42E-36 |
| miR894 | Contig9658 | initiation factor 5a [*Ricinus communis*] | 1.85E-34 |
| miR1507 | AHTC1031528 | unknown [*Glycine max*] | 3.12E-42 |
|  | Contig2318 | predicted protein [*Populus trichocarpa*] | 9.83E-07 |
|  | Contig59 | No hit found |  |
|  | GW966597 | AP2/ERF domain-containing transcription factor [*Populus trichocarpa*] | 2.78E-38 |
|  | HS131_E03 | mucin-related protein [*Arabidopsis thaliana*] | 1.09E-05 |
| miR1511 | AHTC1020531 | No hit found |  |
|  | AHTC1004711 | unknown [*Glycine max*] | 4.53E-120 |
| miR1515 | AHTC1001870 | pectinacetylesterase precursor [*Vigna radiata*] | 0.0 |
|  | AHTC1012040 | No hit found |  |
|  | AHTC1013529 | putative basic helix-loop-helix protein [*Lotus japonicu*s] | 2.08E-47 |
|  | AHTC1014673 | phosphoprotein phosphatase inhibitor | 2.59E-48 |
|  | AHTC1026345 | unknown [*Glycine max*] | 1.07E-67 |
|  | Contig10995 | No hit found |  |
|  | Contig124 | unknown [*Glycine max*] | 1.99E-68 |
|  | Contig17666 | unknown [*Glycine max*] | 2.02E-166 |
|  | Contig30 | unknown [*Glycine max*] | 1.05E-16 |
|  | Contig40 | predicted protein [*Populus trichocarpa*] | 4.23E-61 |
|  | Contig48 | rab5-interacting family protein | 1.15E-10 |
|  | Contig9686 | unknown [*Glycine max*] | 1.58E-89 |
|  | GW946694 | LOB domain-containing protein [*Ricinus communis*] | 6.00E-34 |
| miR2018 | AHTC1002369 | predicted protein [*Populus trichocarp*a] | 1.00E-155 |
| miR2111 | Contig58 | transcription factor jumonji domain-containing protein | 1.32E-18 |
|  | Contig83 | ATP binding protein, putative [*Ricinus communis*] | 3.08E-30 |
| miR2118 | Contig6258 | nitrogenase reductase [*Bradyrhizobium japonicum*] | 3.75E-140 |
| miR2199 | AHTC1035701 | ADP-ribosylation factor GTPase-activating protein AGD2 [*Arabidopsis thaliana* ] | 1.00E-57 |
| miR2910 | AHTC1023139 | No hit found |  |
|  | Contig1038 | aspartate-semialdehyde dehydrogenase [*Glycine max*] | 3.00E-15 |
|  | GW971471 | No hit found |  |
| miR2914 | AHTC1026927 | uncharacterized protein [*Medicago truncatula*] | 5.63E-50 |
|  | Contig11355 | MYB transcription factor MYB85 [*Glycine max*] | 3.92E-47 |
|  | Contig93 | gd2b [*Ricinus communis*] | 1.02E-08 |
|  | Contig94 | endomembrane protein emp70 precusor isolog | 8.70E-38 |
|  | HS073_F08 | chitinase homologue [*Sesbania rostrata*] | 9.00E-09 |
| miR3508 | Contig138 | polyphenol oxidase [*Juglans regia*] | 6.55E-118 |
|  | Contig141 | polyphenol oxidase [*Populus trichocarpa*] | 3.32E-43 |
|  | HS018_A02 | ATP/ADP transporter [*Populus trichocarpa*] | 2.00E-32 |
|  | HS181_A11 | polyphenol oxidase precursor [*Pyrus x bretschneideri*] | 2.00E-52 |
