# Supplementary material for: Identification and Characterization of microRNAs from Peanut (Arachis hypogaea L.) by High-Throughput Sequencing
Source: PLoS One. 2011 Nov 16;6(11):e27530. doi: 10.1371/journal.pone.0027530 (PMC3217988; doi:10.1371/journal.pone.0027530)
Supplement: Table S3 — Identified targets of new miRNAs in peanut. (DOC) [file pone.0027530.s004.doc]

**Table S3.** Identified targets of novel miRNAs in peanut.

| **miRNA family** | **Target EST** | **Annotation** | **E-value** |
| --- | --- | --- | --- |
| miR1 | AHTC1009230 | hypothetical protein [*Oryza sativa*] | 1 |
|  | AHTC1010415 | No hit found |  |
|  | AHTC1035600 | hypothetical protein [*Vitis vinifera*] | 7.00E-26 |
|  | Contig31 | No hit found |  |
|  | Contig5 | No hit found |  |
|  | GW925113 | No hit found |  |
| miR2 | AHTC1001525 | uncharacterized protein [*Glycine max* ] | 4.00E-120 |
|  | AHTC1011431 | chaperone protein dnaJ [*Ricinus communis*] | 1.00E-70 |
|  | AHTC1034108 | No hit found |  |
|  | Contig6139 | pectinesterase [*Cicer arietinum*] | 6.00E-97 |
| miR3 | AHTC1005306 | uncharacterized protein[*Ricinus communis*] | 1.00E-69 |
|  | AHTC1034404 | 1-Deoxy-D-xylulose 5-phosphate synthase 2 [*Medicago truncatula*] | 1.00E-42 |
|  | Contig2957 | No hit found |  |
|  | Contig34 | en spm-like transposon protein [*Ipomoea trifida*] | 1.34E-13 |
| miR4 | AHTC1021021 | No hit found |  |
|  | C20L_004_A05 | unnamed protein product [*Vitis vinifera*] | 8.5 |
|  | Contig128 | elongation factor 1-alpha [*Oryza sativa*] | 1.15E-36 |
|  | Contig129 | elongation factor 1-alpha [*Vitis vinifera*] | 1.34E-05 |
|  | TFR6_004_H05 | unnamed protein product [*Vitis vinifera*] | 8.5 |
| miR5 | AHTC1000353 | Ca+2-binding EF hand protein [*Glycine max*] | 4.00E-114 |
|  | AHTC1011949 | No hit found |  |
|  | Contig1 | No hit found |  |
|  | GW932101 | No hit found |  |
| miR7 | AHTC1018449 | hypothetical protein [*Mycoplasma penetrans*] | 4.6 |
|  | AHTC1009353 | harpin-induced 1 [*Medicago truncatula*] | 1.00E-67 |
|  | Contig10 | No hit found |  |
| miR8 | AHTC1000254 | heat shock protein 90 [*Glycine max*] | 4.00E-37 |
|  | AHTC1019452 | No hit found |  |
|  | AHTC1024892 | No hit found |  |
|  | AHTC1028617 | chlorophyll a/b binding protein [*Solanum lycopersicum*] | 4.00E-90 |
|  | Contig130 | ankyrin repeat-containing [*Vitis vinifera*] | 1.59E-50 |
|  | Contig131 | UDP-glucose 4,6-dehydratase [*Arabidopsis thaliana*] | 3.18E-70 |
|  | Contig137 | conglutin [*Arachis hypogaea*] | 6.49E-36 |
|  | Contig139 | nad-dependent epimerase dehydratase [*Ricinus communis*] | 1.66E-113 |
|  | Contig17589 | nad-dependent epimerase dehydratase [*Glycine max*] | 3.86E-106 |
|  | Contig23 | No hit found |  |
|  | Contig23732 | No hit found |  |
|  | Contig3 | ferric reductase-like transmembrane component [*Medicago truncatula*] | 1.90E-94 |
|  | Contig32 | No hit found |  |
|  | Contig9 | heat shock protein 90-2 [*Glycine max*] | 4.80E-37 |
|  | GO328602 | No hit found |  |
|  | GW967239 | glutathione S-transferase 3 [*Arachis hypogaea*] | 1.86E-54 |
|  | GW967899 | No hit found |  |
|  | GW975624 | unknown [*Glycine max*] | 6.37E-28 |
|  | GW990367 | uncharacterized protein [*Glycine max*] | 1.00E-28 |
|  | HS019_H02 | ankyrin repeat-containing protein [*Ricinus communis*] | 2.00E-49 |
| miR9 | Contig132 | No hit found |  |
|  | GW939963 | No hit found |  |
| miR10 | C20L_004_A05 | unnamed protein product [*Vitis vinifera*] | 8.5 |
|  | Contig128 | elongation factor 1-alpha [*Oryza sativa*] | 1.15E-36 |
|  | Contig129 | elongation factor 1-alpha [*Vitis vinifera*] | 1.34E-05 |
|  | TFR6_004_H05 | unnamed protein product [*Vitis vinifera*] | 8.5 |
| miR11 | AHTC1003832 | ATP binding protein [*Ricinus communis*] | 1.00E-109 |
| miR12 | GW974237 | No hit found |  |
| miR13 | Contig10374 | No hit found |  |
|  | Contig14 | cc-nbs-lrr resistance protein [*Arachis cardenasii*] | 2.90E-48 |
|  | Contig14310 | No hit found |  |
|  | Contig2037 | No hit found |  |
|  | Contig9422 | cold induced plasma membrane protein [*Jatropha curcas*] | 1.00E-15 |
| miR14 | AHTC1000805 | thioredoxin f-type [*Ricinus communis*] | 5.00E-55 |
|  | AHTC1001095 | single-stranded nucleic acid binding R3H [*Medicago truncatula*] | 1.00E-139 |
|  | AHTC1003005 | unknown [*Glycine max*] | 4.00E-149 |
|  | AHTC1007505 | Photosystem II reaction center PsbP family protein [*Arabidopsis thaliana*] | 1.00E-97 |
|  | AHTC1007956 | ATP binding protein [*Ricinus communis*] | 6.00E-21 |
|  | AHTC1008882 | sbp (s-ribonuclease binding protein) family protein [*Populus trichocarpa*] | 1.32E-10 |
|  | AHTC1019739 | No hit found |  |
|  | AHTC1021122 | putative glycosyltransferase [*Clitoria ternatea*] | 9.00E-74 |
|  | AHTC1029097 | sec23/sec24-like transport protein [*Arabidopsis thaliana*] | 3.00E-47 |
|  | AHTC1030042 | transcription factor homolog BTF3-like protein [*Lotus japonicus*] | 3.00E-62 |
|  | AHTC1031662 | UDP-glucuronate 4-epimerase [*Arabidopsis thaliana*] | 5.00E-45 |
|  | AHTC1034584 | GTP cyclohydrolase II, putative [*Ricinus communis*] | 1.00E-54 |
|  | AHTC1034740 | No hit found |  |
|  | AHTC1035704 | tRNA-binding arm; t-snare [*Medicago truncatula*] | 2.00E-11 |
|  | AHTC1036290 | diacylglycerol kinase family protein [*Arabidopsis lyrata*] | 2.00E-45 |
|  | AHTC1036643 | uncharacterized protein [*Glycine max*] | 2.00E-44 |
|  | AHTC1036672 | gtp binding protein [*Glycine max*] | 4.98E-68 |
|  | Contig11 | No hit found |  |
|  | Contig133 | leucine-rich repeat family protein [*Vitis vinifera*] | 7.86E-27 |
|  | Contig14748 | DIM-like protein [*Glycine max*] | 2.00E-12 |
|  | Contig15 | peroxisomal ascorbate peroxidase [*Glycine max*] | 1.95E-46 |
|  | Contig16 | unknown [*Glycine max*] | 2.61E-54 |
|  | Contig17 | phenylalanyl-trna synthetase [*Populus trichocarpa*] | 8.36E-34 |
|  | Contig19 | protease complex subunit 3 [*Glycine max*] | 1.97E-10 |
|  | Contig19976 | uncharacterized protein [*Ricinus communis*] | 8.00E-10 |
|  | Contig20 | translation elongation factor tu [*Ricinus communis*] | 6.19E-43 |
|  | Contig21 | predicted protein [*Populus trichocarpa*] | 1.24E-05 |
|  | Contig21056 | UDP-D-glucuronate 4-epimerase 4 [*Arabidopsis thaliana*] | 1.00E-19 |
|  | Contig26 | No hit found |  |
|  | Contig26409 | poz btb containing-protein 1 | 1.09E-54 |
|  | Contig27 | vesicle-associated membrane family protein | 4.17E-85 |
|  | Contig30 | unknown [*Glycine max*] | 1.05E-16 |
|  | Contig4191 | fiber protein Fb14 [*Gossypium barbadense*] | 2.28E-39 |
|  | Contig7146 | unnamed protein product [*Vitis vinifera*] | 7.15E-47 |
|  | Contig8 | monoterpene glucosyltransferase | 1.52E-16 |
|  | Contig8697 | 3-5-exoribonuclease family protein | 3.78E-116 |
|  | Contig88 | No hit found |  |
|  | HS030_A12 | No hit found |  |
| miR16 | AHTC1032986 | No hit found |  |
|  | AHTC1018300 | aldo/keto reductase [*Ricinus communis*] | 6.00E-40 |
|  | AHTC1010143 | cytokinin oxidase [*Populus trichocarpa*] | 4.41E-12 |
|  | AHTC1009230 | hypothetical protein [*Oryza sativa*] | 1 |
|  | AHTC1008109 | unknown [*Glycine max*] | 8.38E-11 |
|  | Contig13650 | No hit found |  |
| miR17 | AHTC1030212 | nitrate transporter [*Nicotiana tabacum*] | 6.00E-69 |
|  | AHTC1032501 | calcium-dependent protein | 1.58E-10 |
|  | Contig134 | nitrate transporter [*Nicotiana tabacum*] | 7.72E-69 |
|  | Contig135 | unknown [*Glycine max*] | 4.41E-24 |
|  | Contig14754 | No hit found |  |
|  | Contig1674 | No hit found |  |
|  | Contig18 | formin-like protein AHF1 [*Arabidopsis thaliana*] | 5.57E-14 |
|  | Contig2 | No hit found |  |
|  | Contig22 | fimbrin [*Ricinus communis*] | 4.12E-19 |
|  | Contig22590 | kinesin-3 [*Ricinus communis*] | 3.00E-14 |
|  | Contig2664 | predicted protein [*Populus trichocarpa*] | 2.04E-11 |
|  | Contig33 | atp-dependent rna helicase dhh1 | 4.56E-75 |
|  | Contig4349 | translation releasing factor 2 [*Cucumis sativus*] | 1.00E-65 |
|  | Contig4815 | centromere specific histone H3 variant [*Nicotiana tabacum*] | 1.80E-21 |
|  | GW937782 | predicted protein [*Populus trichocarpa*] | 1.19E-15 |
|  | HS190_A12 | formin-like protein AHF1 [*Arabidopsis thaliana*] | 3.00E-26 |
|  | HS254_C10 | nitrate transporter [*Nicotiana tabacum*] | 8.00E-69 |
|  | HS263_G04 | formin-like protein 1 [*Arabidopsis thaliana*] | 4.00E-31 |
| miR18 | Contig7 | No hit found |  |
| miR19 | AHTC1014981 | epoxide hydrolase | 1.66E-48 |
|  | Contig12 | retrotransposon gag protein [*Asparagus officinalis*] | 7.21E-68 |
|  | Contig25957 | No hit found |  |
| miR20 | Contig4 | hypothetical protein [*Vitis vinifera*] | 1.97E-07 |
|  | GW976637 | No hit found |  |
| miR21 | AHTC1033405 | No hit found |  |
|  | Contig4707 | lectin-like protein [*Medicago truncatula*] | 3.40E-16 |
| miR22 | AHTC1002952 | histone deacetylase [*Populus trichocarpa*] | 0 |
|  | AHTC1026049 | gag-pol polyprotein [*Glycine max*] | 1.00E-24 |
|  | AHTC1036548 | unknown protein [*Arabidopsis thaliana*] | 1.00E-11 |
|  | Contig11849 | hypothetical protein [*Ricinus communis*] | 1.46E-11 |
| miR23 | AHTC1026134 | hypothetical protein [*Vitis vinifera*] | 0.011 |
|  | AHTC1030494 | unknown [*Glycine max*] | 1.98E-23 |
|  | Contig13 | No hit found |  |
|  | Contig19846 | uncharacterized protein [*Medicago truncatula*] | 6.00E-10 |
|  | Contig25 | acyl-activating enzyme 18 | 1.17E-13 |
|  | Contig9925 | hypothetical protein [*Beta vulgaris*] | 1.64E-04 |
|  | GW976210 | No hit found |  |
| miR24 | Contig143 | 40s ribosomal protein s5 | 7.26E-68 |
|  | Contig2212 | aspartate carbamoyltransferase | 3.63E-102 |
|  | Contig24 | protein phosphatase type 2c | 7.63E-29 |
|  | GW974788 | synaptonemal complex protein [*Ricinus communis*] | 3.37E-04 |
| miR25 | Contig22708 | signal recognition particle 68 kda | 3.79E-12 |
|  | Contig29 | No hit found |  |
